# Vitamin D Metabolite Profile in Cholecalciferol- or Calcitriol-Supplemented Healthy and Mammary Gland Tumor-Bearing Mice

**DOI:** 10.3390/nu12113416

**Published:** 2020-11-06

**Authors:** Artur Anisiewicz, Konrad Kowalski, Joanna Banach, Natalia Łabędź, Martyna Stachowicz-Suhs, Aleksandra Piotrowska, Magdalena Milczarek, Dagmara Kłopotowska, Piotr Dzięgiel, Joanna Wietrzyk

**Affiliations:** 1 Department of Experimental Oncology, Hirszfeld Institute of Immunology and Experimental Therapy, 53-114 Wroclaw, Poland; artur.anisiewicz@hirszfeld.pl (A.A.); joanna.banach@hirszfeld.pl (J.B.); natalia.labedz@hirszfeld.pl (N.Ł.); martyna.stachowicz@hirszfeld.pl (M.S.-S.); magdalena.milczarek@hirszfeld.pl (M.M.); dagmara.klopotowska@hirszfeld.pl (D.K.); 2 Research and Development Center Masdiag, 01-882 Warsaw, Poland; konrad.kowalski@mas-diag.pl; 3 Department of Histology and Embryology, Faculty of Medicine, Wroclaw Medical University, 50-368 Wroclaw, Poland; aleksandra.piotrowska@umed.wroc.pl (A.P.); piotr.dziegiel@umed.wroc.pl (P.D.); 4 Department of Physiotherapy, Wroclaw University School of Physical Education, 51-612 Wroclaw, Poland

**Keywords:** calcitriol, cholecalciferol, vitamin D deficiency, breast cancer, vitamin D metabolites, 25(OH)D_3_, 24,25(OH)_2_D_3_, 3-epi-25(OH)D_3_, BALB/c, C57BL/6

## Abstract

To analyze if the prometastatic activity of calcitriol (active vitamin D_3_ metabolite), which was previously observed in a 4T1 breast cancer model, is also found in other breast cancers, and to assess the impact of various schemes of vitamin D supply, we used 4T1 and E0771 mouse metastatic and 67NR nonmetastatic cells in this study. BALB/c and C57BL/6 healthy and tumor-bearing mice were exposed to a control (1000 IU), low- (100 IU), and high- (5000 IU) vitamin D_3_ diets. Additionally, from day 7 of tumor transplantation, the 1000 and 100 IU groups were gavaged with calcitriol (+cal). After 8 weeks of feeding, plasma levels of 25(OH)D_3_, 24,25(OH)_2_D_3_, and 3-epi-25(OH)D_3_ were significantly lower in calcitriol-treated and vitamin D-deficient groups than in the control, whereas the levels of all metabolites were increased in the 5000 IU group. The ratio of 25(OH)D_3_:24,25(OH)_2_D_3_ was increased in both calcitriol-treated groups, whereas the ratio of 25(OH)D_3_:3-epi-25(OH)D_3_ was increased only in the 100 IU group but decreased in the 5000 IU group. In contrast to E0771, 4T1 lung metastasis was accelerated in all vitamin D-supplemented mice, as well as in the deficient group with an increased inflammatory response. 67NR tumor growth was transiently inhibited in the 1000 IU+cal group, but single metastases were observed in the 5000 and 100 IU groups. Based on the results, we conclude that various schemes of vitamin D supply and vitamin D deficiency led to similar metabolite profiles irrespective of the mice strain and tumor burden. However, depending on the type of breast cancer, different effects on tumor growth and metastasis were noticed.

## 1. Introduction

A low level of vitamin D, indicating a low level of 25-hydroxy vitamin D (25(OH)D), and low level of vitamin D receptor (VDR) in tumor tissue are recognized as risk factors of breast cancer and correlated with clinical parameters such as the incidence of breast cancer, tumor biology, prognosis, and treatment tolerance [1,2,3,4,5,6]. After adjuvant therapy, vitamin D deficiency becomes severe in breast cancer patients, altering their bone metabolism and increasing their risk of osteoporosis [7,8]. Therefore, correction of vitamin D deficiency has been suggested as a potential strategy to delay recurrence and extend the survival of these patients [9,10]. However, a recent meta-analysis has shown the significant protective effect of high levels of serum 25(OH)D only in premenopausal patients [11]. Moreover, the results of a randomized, placebo-controlled trial, engaging a large number of participants and lasting for 5 years, have indicated that vitamin D supplementation (2000 IU) had no effect on the incidence of invasive cancer (including breast cancer) [12]. On the other hand, some evidence for an increase in the risk of breast cancer with higher 25(OH)D levels was provided by a European population-based cohort study conducted among older adults [13]. Recently, Kanstrup et al. also reported that women with high levels of 25(OH)D have poorer breast cancer survival [14].

Experimental data obtained with the use of xenografts of human breast cancer cell lines [15] and allografted mouse tumors [16,17,18] showed that vitamin D deficiency promotes tumor growth and metastasis. The anticancer and antimetastatic activities of calcitriol (1,25(OH)_2_D_3_, active metabolite of vitamin D_3_) or its analogs have been observed in various breast cancer models [19,20,21]. On the other hand, in our recently published study, we presented that calcitriol (and its analogs) can accelerate metastasis in the premenopausal model of 4T1 mouse mammary gland cancer, even when the cancer cells were not sensitive to its activity, thus influencing the cancer microenvironment [22]. However, contrary to our results, Zhang et al. showed the inhibition of metastatic processes after calcitriol treatment in the same tumor model [23]. It should be noted that in our experiment, a calcitriol dose was given on day 7 after the implantation of tumor cells, when tumors became palpable [22], whereas in the experiment conducted by Zhang et al., the treatment was started one day before tumor cell transplantation [23]. Moreover, another study has indicated that the growth of primary 4T1 tumor was accelerated upon calcitriol treatment beginning 17 days after the inoculation of tumor cells [24]. Therefore, it seems that the final effect of calcitriol or cholecalciferol administration may depend on the stage of tumor development when the treatment was initiated.

Despite many years of research on the use of calcitriol or its analogs for cancer treatment, it has not yet been possible to introduce them into clinical practice and clinical trials did not provide the expected results [25]. Diet supplementation with vitamin D, on the other hand, is widely recommended, and studies are being conducted to confirm its benefits in cancer patients [2]. Cholecalciferol (vitamin D_3_) supplementation is rather safe to prevent the development of hypercalcemia, because vitamin D_3_ is metabolized in the body to active calcitriol and the mechanisms that maintain homeostasis and prevent toxicity [26,27]. Jeong et al. and other groups of authors showed similar effects on the development of mammary gland tumors when a vitamin D-supplemented diet was given to mice or calcitriol was injected [27,28]. These observations, along with the results of other studies, suggest the possibility that dietary vitamin D, rather than the active hormone calcitriol, could be used in breast cancer therapy [27]. In some of these studies, the levels of 25(OH)D and/or 1,25(OH)_2_D_3_ were controlled and the mRNA expression of cytochrome P450 enzymes involved in vitamin D metabolism, mainly 1α-hydroxylase CYP27B1, as well as calcium levels, were evaluated [22,27,28,29,30]. The decrease in circulating 25(OH)D after treatment with calcitriol or its elevation by dietary vitamin D was also analyzed in these studies [22,27,29,30].

However, there are limited experimental data regarding vitamin D metabolism (calcitriol vs. dietary vitamin D) in tumor-bearing mice and in healthy counterparts, especially regarding the analysis of vitamin D_3_ epimers. Therefore, the aim of the present study was to trace the metabolic profile of vitamin D in two different strains of healthy and tumor-bearing female mice. For this purpose, metastatic and nonmetastatic, well-defined mouse mammary gland tumors were transplanted to mice exposed to a normal, low-, and high-cholecalciferol diet, and the mice were additionally treated with calcitriol when tumors became palpable.

## 2. Materials and Methods

### 2.1. Mice

BALB/c and C57BL/6 female, 6- to 8-week-old mice, weighing 20–25 g, were obtained from Charles River Laboratories (Sulzfeld, Germany). The animals were maintained in specific pathogen-free conditions.

All the experiments were performed in accordance with the EU Directive 2010/63/EU on the protection of animals used for scientific purposes and were approved by the first Local Committee for Experiments with the Use of Laboratory Animals, Wroclaw, Poland (permission number: 66/2018).

### 2.2. Cell Lines

The mouse mammary adenocarcinoma 4T1 cells were obtained from the American Type Culture Collection (ATCC, Rockville, MD, USA), and 67NR (nonmetastatic counterparts of 4T1) cells were obtained from Barbara Ann Karmanos Cancer Institute (Detroit, MI, USA). The E0771 cell line [31] was kindly gifted by Dr. Andreas Möller (School of Medicine, University of Queensland; Tumour Microenvironment Laboratory, QIMR Berghofer Medical Research Institute, Herston, Queensland, Australia).

The 4T1 cell line was maintained in RPMI-1640—GlutaMAX (Thermo Fisher Scientific, Waltham, MA, USA)—with 10% fetal bovine serum (FBS) HyClone (GE Healthcare, Chicago, IL, USA) adjusted to contain 3.5 g/L glucose and 1.0 mM sodium pyruvate (Sigma-Aldrich Chemie GmbH, Steinheim, Germany). 67NR cells were cultured in Dulbecco’s modified Eagle’s medium (DMEM; Gibco, Scotland, UK) containing 10% calf bovine serum (ATCC, Rockville, MD, USA), 1% amino acid, and 2 mM L-glutamine (Sigma-Aldrich Chemie GmbH, Steinheim, Germany). The E0771 cell line was cultured in DMEM (Gibco, Scotland, UK) containing 10% FBS (GE Healthcare, Chicago, IL, USA), and 2 mM L-glutamine (Sigma-Aldrich Chemie GmbH, Steinheim, Germany). All the culture media were supplemented with 100 U/mL of penicillin (Polfa Tarchomin S.A., Warsaw, Poland) and 100 µg/mL of streptomycin (Sigma-Aldrich Chemie GmbH, Steinheim, Germany). The cells were grown at 37 °C in a humid atmosphere saturated with 5% CO_2_.

### 2.3. Tumor Cell Transplantation, Tumor Growth and Metastasis, Diet Composition, and Calcitriol Administration

**Figure 1:**
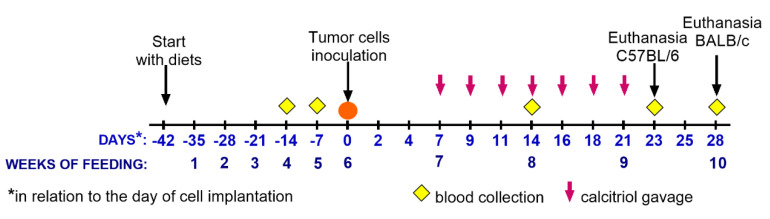
Time-course of experiments conducted in BALB/c and C57BL/6 tumor-bearing mice. Euthanasia and the last blood collection in the case of C57BL/6 mice were performed on day 23.

The mice were kept on a synthetic diet AIN67 (ZooLab, Sedziszow, Poland) for 6 weeks. The diet had controlled contents of vitamin D_3_ as follows: normal amount of vitamin D (1000 IU; control), high amount of vitamin D (5000 IU; supplementation), and low amount of vitamin D (100 IU; deficient). At weeks 4 (BALB/c) and 5 (BALB/c and C57BL/6) of feeding, blood was collected from the zygomatic vein of mice and the level of vitamin D_3_ metabolites was analyzed. After 6 weeks—on day 0—some mice were implanted with tumor cells (as described below), and the diets were continued. Simultaneously, 7 days after the implantation of tumor cells, calcitriol (1 µg/kg of body weight, per os by gavage) was administered. Calcitriol administration was carried out three times a week in the groups receiving food with a normal level of vitamin D_3_ and in the groups receiving the vitamin D-deficient diet (summarized in Figure 1 and Table 1).

Each BALB/c or C57BL/6 female mouse was orthotopically inoculated with 1 × 10^4^, 2 × 10^5^, and 5 × 10^4^ viable 4T1, 67NR, and E0771 tumor cells in 0.05 mL Hanks solution, respectively, in the right mammary fat pad (Table 1).

On days -14 (4 weeks), -7 (5 weeks), and 14 (days counted in reference to the day of tumor inoculation which was assigned as day 0; weeks counted from the start of experimental diets), blood was collected from the mice by puncturing the zygomatic vein. On day 23 (C57BL/6) or 28 (BALB/c) of cancer cell inoculation, blood was harvested from the mice under isoflurane (Aerrane Isofluranum, Baxter, Canada) anesthesia, and then buprenorphine solution at a dose of 0.1 mg/kg of body weight was injected subcutaneously and the mice were euthanized. Subsequently, the lungs, tumors, livers, and kidneys were harvested for further analyses (Figure 1).

Tumor volume was calculated using the formula (*a*^2^ × *b*)/2, where *a* is the shorter tumor diameter in millimeters and *b* is the longer tumor diameter in millimeters. Body weight of the mice was monitored throughout the experiment.

A blinded macroscopic count of metastatic foci was performed on the surface of lungs fixed with 4% paraformaldehyde. Overnight fixation of the lungs allowed to distinguish between metastatic foci and lung tissue. Lungs were placed under a dissecting stereomicroscope and the number of metastases visible on each lobe of the lung was counted. The hematoxylin and eosin (H&E)-stained slides were evaluated under a BX-41 light microscope (Olympus, Tokyo, Japan) by two independent pathologists to assess large metastatic foci and disseminated metastases. Large metastatic foci were identified as clusters of neoplastic cells with a diameter greater than 2 mm, while disseminated metastases were considered as clusters of cancer cells with dimensions of 0.2–2 mm.

### 2.4. Measurements of Vitamin D Metabolites

The vitamin D metabolite profile (25(OH)D_3_, 24,25(OH)_2_D_3_, and epi-25(OH)D_3_) was analyzed by high-performance liquid chromatography coupled with mass spectrometry following an isotope dilution methodology. Whole blood was collected on Whatman 903 cards, and dried blood spots were prepared. Two 3-mm discs were cut out, pooled together, and subjected to methanol extraction. Before analysis, vitamin D metabolites were derivatized with 4-(4′-dimethylaminophenyl)-1,2,4-triazoline-3,5-dione (DAPTAD) [32]. Chromatographic separation (Exion LC, Sciex) was performed on a Kinetex 1.7 µm F5 100 Å, 50 × 2.1 mm column in 8-min gradients of water and acetonitrile with 0.1% formic acid (0.45 mL/min; 40 °C). Detection was conducted using an MRM technique on 4500QTRAP (Exion LC, Sciex) in the electrospray positive ionization mode. The results were multiplied by hematocrit values to determine the serum concentrations of vitamin D metabolites [33].

### 2.5. Western Blot Analysis of CYP2R1, CYP27A1, CYP24, and VDR

#### 2.5.1. Tissue Lysate Preparation

Tumors, livers, and kidneys were frozen in liquid nitrogen and stored at –80 °C. Samples were prepared from frozen tissue and subsequently transferred to tubes containing homogenizing ball (Mp Biomedicals LLC., Santa Ana, CA, USA) and radioimmunoprecipitation assay (RIPA) buffer with a cocktail of phosphatase and protease inhibitors (Sigma-Aldrich Chemie GmbH, Steinheim, Germany). Homogenization was carried out using a Fast Prep^®^-24 MP Bio homogenizer (Mp Biomedicals LLC., Santa Ana, CA, USA) as described previously [22]. The concentration of proteins in the homogenized sample was determined using a DC Protein assay (Bio-Rad, Hercules, CA, USA), according to the manufacturer’s instructions.

#### 2.5.2. Western Blot Analyses

Briefly, polyacrylamide gel electrophoresis was carried out on 50 μg of protein samples. The proteins were transferred onto polyvinylidene difluoride (PVDF) membranes with a pore size of 0.45 μm (Merck Millipore, Billerica, MA, USA). After incubating for 1 h with 5% nonfat dry milk in 0.1% Tris-buffered saline/Tween-20 (Hirszfeld Institute of Immunology and Experimental Therapy, Polish Academy of Sciences (HIIET PAS), Wroclaw, Poland/Sigma-Aldrich, Saint-Louis, MO, USA), the membranes were probed with the following antibodies (in corresponding dilutions) overnight at 4 C: rabbit anti-VDR monoclonal antibody (1:1000; D2K6W; Cell-Signaling, Danvers, MA, USA); rabbit anti-CYP24A1 polyclonal antibody (1:1000; H087, #sc-66851; Santa Cruz Biotechnology, Dallas, TX, USA); rabbit anti-CYP27B1 monoclonal antibody (1:1000; ab206655; Abcam, Cambridge, UK); rabbit anti-CYP2R1 polyclonal antibody (1:2000; ab137634; Abcam, Cambridge, UK). On the next day, the membranes were washed and incubated with secondary mouse antirabbit immunoglobulin G–horseradish peroxidase (HRP) monoclonal antibody (1:10,000; Santa Cruz Biotechnology Inc., Dallas, TX, USA) for 1 h. Chemiluminescence was induced using Clarity Western ECL Substrate (Bio-Rad, Hercules, CA, USA), and detection was performed on a ChemiDoc Imaging System (Bio-Rad, Hercules, CA, USA). Subsequently, the membranes were incubated for 30 min with 100% methanol at room temperature (RT; Avantor Performance Materials Poland, Gliwice, Poland) to remove the bound antibodies. Then, the membranes were washed, blocked for 1 h, washed again, and incubated with mouse anti-β-actin-HRP (C4) monoclonal antibody (1:1000; Santa Cruz Biotechnology Inc., Dallas, TX, USA) for 1 h at RT. Detection of proteins was performed as described above. Densitometry analysis was carried out in ImageJ software with the tested protein normalized to β-actin.

### 2.6. Immunohistochemical Staining for VDR Expression of Lung and Tumor Tissue

All immunohistochemical reactions were performed on 4-µm-thick paraffin sections using Autostainer Link48 (Dako, Glostrup, Denmark). Deparaffinization, rehydration, and epitope retrieval were carried out in EnVision FLEX Target Retrieval Solution High pH (97 °C, 20 min) in PT-Link (both from Dako). To inactivate endogenous peroxidase, the slides were incubated for 5 min with EnVision FLEX Peroxidase-Blocking Reagent (Dako). Subsequently, the sections were incubated first with primary antibody against vitamin D_3_ receptor (clone D2K6W, dilution 1:200, cat. no. 12550S; Cell Signaling Technology, Danvers, MA, USA) for 20 min at RT and then with secondary antibody (EnVision FLEX/HRP) for 20 min (Dako). The substrate for peroxidase, 3,3′-diaminobenzidine (Dako), was applied, and the sections were incubated at RT for 10 min. Finally, the slides were counterstained with EnVision FLEX Hematoxylin for 5 min (Dako), dehydrated in a graded series of ethanol and xylene, and mounted using SUB-X Mounting Medium (Dako).

The intensity of the expression of VDR antigen in tumor cells was evaluated by determining the proportion of positive cells among the tumor cells: 0 points—absence of the reaction; 1—1–10% positive cells; 2—11–25% positive cells; 3—26–50% positive cells; 4—over 50% of the cells showed a positive reaction.

### 2.7. Plasma Biochemical Parameters

The levels of calcium, phosphate, creatinine, total protein, albumin, and alkaline phosphatase were measured in each individual plasma sample using Cobas c 111 with ISE (Roche Diagnostics Ltd., Rotkreuz, Switzerland).

### 2.8. Blood Morphology

Whole blood was collected on Heparinum WZF (Polfa Tarchomin S.A., Warsaw, Poland) at 5000 IU/mL (100 µL of heparin solution per sample) and analyzed using a Mythic 18 hematology analyzer (C2 Diagnostics, Montpellier, France).

### 2.9. Statistical Analysis

Statistical analysis was performed using GraphPad Prism 7.1 software. The normality of the data distribution was analyzed using the Shapiro–Wilk data normality test (significance of the test was assumed at *p* < 0.05). Based on the distribution, a statistical analysis of individual data is presented in the figure legends. Differences between the groups at *p* < 0.05 were considered statistically significant.

## 3. Results

### 3.1. Body Weight and Tumor Growth Kinetics in BALB/c and C57BL/6 Mice

**Figure 2:**
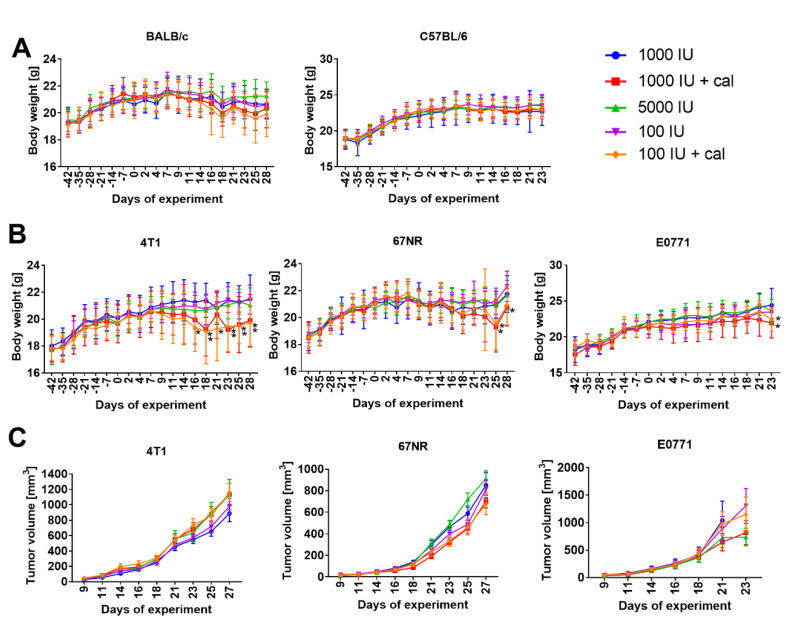
Body weight as well as tumor growth kinetics in BALB/c and C57BL/6 tumor-bearing mice. (**A**) Body weight of healthy BALB/c and C57BL/6 female mice. (**B**) Body weight of tumor-bearing BALB/c (4T1 and 67NR) and C57BL/6 (E0771) female mice. (**C**) Kinetics of tumor growth in BALB/c (4T1 and 67NR) and C57BL/6 (E0771) mice. N = 10–12 mice/group (BALB/c) or 7–12 mice/group (C57BL/6); several mice bearing E0771 tumors were euthanized when tumors reached 2000 mm^3^ because of ethical reasons, and thus, there were different numbers of mice at subsequent time-points. Mean values and (**A**,**B**) standard deviation or (**C**) standard error of mean are presented. Statistical analysis: Sidak’s multiple comparisons test. * *p* < 0.05 100 IU+cal as compared to 1000 IU; ** *p* < 0.05 1000 IU+cal and 100 IU+cal as compared to 1000 IU.

The body weight of healthy BALB/c and C57BL/6 mice was not found to be significantly affected by the diets used or by calcitriol administration (Figure 2A). However, in aggressive 4T1 tumor-bearing BALB/c mice administered with calcitriol and fed a diet containing normal level of cholecalciferol (1000 IU) or with a vitamin D-deficient diet (100 IU), significant weight loss was observed on days 18, 23–28 (1000 IU+cal) or 16–28 (100 IU+cal) (Figure 2B). Less aggressive 67NR tumors also contributed to the loss of body weight in mice administered with calcitriol; significant weight loss was observed on days 25 and 28 in 100 IU+cal group and on day 25 in the 1000 IU+cal group (Figure 2B). Among E0771 tumor-bearing C57BL/6 mice, for which the monitoring time was shortened because of excessive tumor growth during the last time-point of observation, statistically significant weight loss was observed in both groups receiving calcitriol (Figure 2B).

The diets and treatments used did not seem to significantly affect the growth of 4T1 or E0771 tumors, but during the growth of 67NR transient antitumor effects were observed (Figure 2C). For instance, on day 23, mice from the 1000 IU+cal group had a significantly lower tumor volume as compared to the control group (1000 IU) (*p* < 0.05; 325 ± 83 mm^3^ vs. 468 ± 197 mm^3^, respectively). On day 25, the change in tumor volume was less pronounced (*p* = 0.0512) between the 1000 IU+cal group and the control group (1000 IU) (450 ± 144 mm^3^ and 587 ± 211 mm^3^, respectively). On the subsequent days, no significant effects of applied diets/treatments were found on the growth of 67NR tumors.

**Figure 3:**
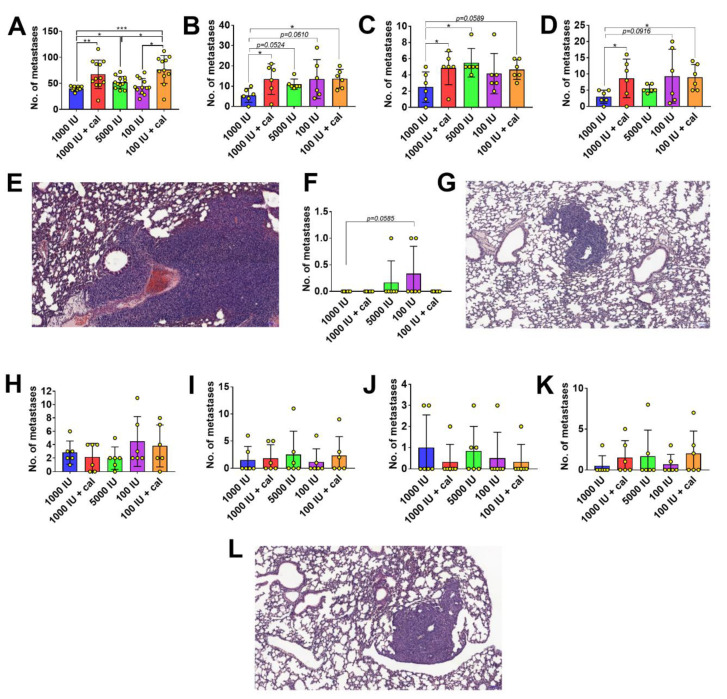
Macroscopic and histopathological analyses of lung metastases. (**A**–**E**) 4T1: (**A**) macroscopic count for metastases; (**B**) histopathological count for metastases—sum of large metastatic foci and disseminated metastases; (**C**) no. of histologically determined large metastatic foci; (**D**) no. of disseminated metastases; (**E**) representative hematoxylin and eosin (H&E) staining of the lungs from 4T1 tumor-bearing mice; magnification—×100, scale bar—100 µm. (**F**,**G**) 67NR: (**F**) histopathological analysis—single metastatic foci; (**G**) representative H&E staining of the lungs from 67NR tumor-bearing mice; magnification—×100, scale bar—100 µm. (**H**–**L**) E0771: (**H**) macroscopic count for metastases; (**I**) histopathological count for metastases—sum of large metastatic foci and disseminated metastases; (**J**) no. of histologically determined large metastatic foci; (**K**) no. of disseminated metastases; (**L**) representative H&E staining of the lungs from E0771 tumor-bearing mice; magnification—×100, scale bar—100 µm. Mean values and standard deviation with data for individual mice are presented. Statistical analysis: Dunn’s multiple comparisons test or (**A**) and (**H**) Sidak’s multiple comparisons test. * *p* < 0.05; ** *p* < 0.01; *** *p* < 0.001.

The macroscopic count of metastatic foci revealed a significant increase in the number of metastases in the lung of mice bearing 4T1 tumors gavaged with calcitriol, fed with diets containing 1000 IU or 100 IU of cholecalciferol (1000 IU+cal and 100 IU+cal). An increased number of metastases was also observed in mice fed with 5000 IU cholecalciferol (Figure 3A). This observation was confirmed by histopathological examination, especially when we analyzed all metastatic lesions in the lungs (Figure 3B) or only the large metastatic foci (Figure 3C). However, histopathological examination also showed an increased number of lesions in mice fed with the vitamin D_3_-deficient diet (100 IU; Figure 3B,D). Macroscopically, we did not detect any metastases in the lungs of mice bearing 67NR tumors; however, in the histopathological analysis, we observed a single metastatic foci in one mouse fed with a vitamin D-supplemented diet (5000 IU) and in two mice fed with the deficient diet (100 IU; Figure 3F,G). C57BL/6 mice bearing E0771 tumors had a similar number of metastases in the lung independent of treatment. Only mice fed with the deficient diet showed a tendency toward increased lung metastasis, and treatment of these mice with calcitriol did not produce any effect on their metastases (*p* = 0.4; Figure 3H); however, this was not confirmed by the histopathological examination (Figure 3I–L).

### 3.2. Vitamin D Metabolite Plasma Levels in Healthy Mice

**Figure 4:**
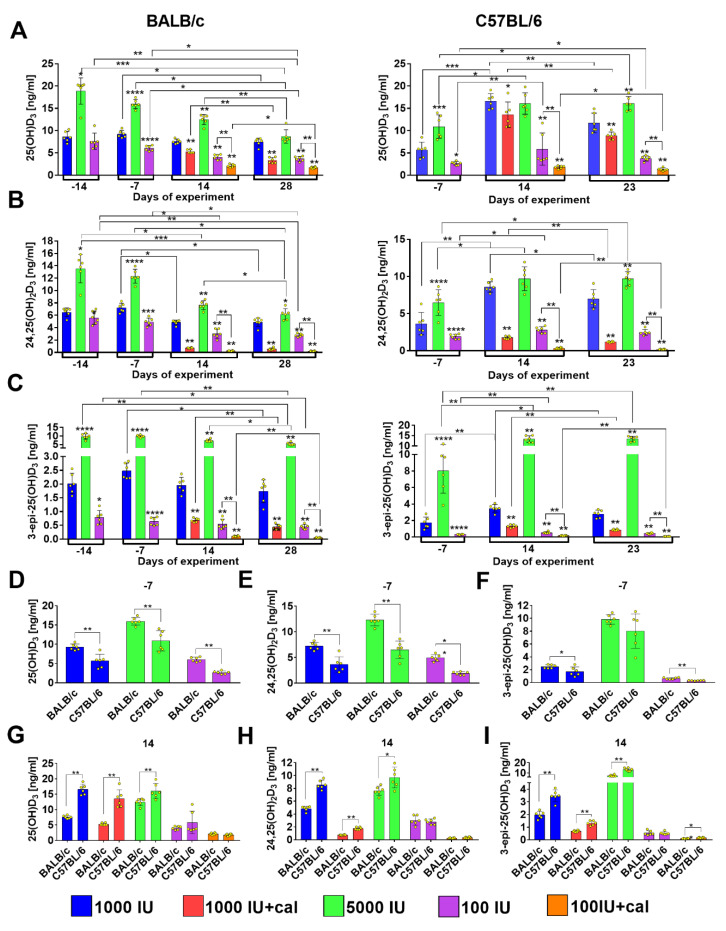
Kinetics of vitamin D metabolite plasma level in healthy BALB/c and C57BL/6 mice. (**A**) 25(OH)D_3_, (**B**) 24,25(OH)_2_D_3_, and (**C**) 3-epi-25(OH)D_3_ plasma level in BALB/c (left column) and C57BL/6 (right column) mice. (**D**–**I**) Comparison of plasma levels of vitamin D metabolites between BALB/c and C57BL/6 mice: on day -7 (5 weeks of experimental diet feeding)—(**D**) 25(OH)D_3_, (**E**) 24,25(OH)_2_D_3_, and (**F**) 3-epi-25(OH)D_3_; on day 14 (8 weeks of experimental diet feeding)—(**G**) 25(OH)D_3_, (**H**) 24,25(OH)_2_D_3_, and (**I**) 3-epi-25(OH)D_3_. Data are presented as mean with standard deviation; individual data are also presented as yellow points. Plasma from 6 mice/group was analyzed. Statistical analysis: Dunn’s test for multiple comparisons or Mann–Whitney test. * *p* < 0.05; ** *p* < 0.01; *** *p* < 0.001; **** *p* < 0.0001.

In BALB/c mice, 4 weeks of feeding (described as day -14 on the graphs, namely 14 days before the day of tumor cell transplantation) with the 5000 IU vitamin D diet was enough to induce a statistically significant increase in the plasma levels of 25(OH)D_3_ (Figure 4A), 24,25(OH)_2_D_3_ (Figure 4B), and 3-epi-25(OH)D_3_ (Figure 4C), but the deficient diet (100 IU) was to be fed for 5 weeks (described as day -7 on the graph) in order to cause a significant decrease in 25(OH)D_3_ and 24,25(OH)_2_D_3_. Only 3-epi-25(OH)D_3_ was significantly decreased after 4 weeks (Figure 4C). Therefore, in C57BL/6 mice, the measurements were taken from the fifth week of feeding, which also resulted in statistically significant changes in the plasma level of 25(OH)D_3_ in mice fed with the diet supplemented with or deficient in vitamin D (Figure 4A–C). From the eighth week (described as day 14 on the graph) of feeding, half of the mice on the control and deficient diets were supplemented with calcitriol. Regardless of whether the mice were fed a control fodder or the diet deficient in vitamin D, calcitriol statistically significantly reduced the levels of 25(OH)D_3_ (Figure 4A), 24,25(OH)_2_D_3_ (Figure 4B), and 3-epi-25(OH)D_3_ (Figure 4C) in both strains and the decrease was observed on both days of measurement (days 14 and 28 in BALB/c or 14 and 23 in C57BL/6). Interestingly, all the tested metabolites increased significantly until week 5 (BALB/c) or 8 (C57BL/6) in mice fed with the supplemented diet (5000 IU), and then started to decrease, which was especially noticeable in BALB/c mice (Figure 4A–C, left panel). It seems that with increasing age (and/or with prolonged supplementation or deficit of vitamin D) the levels of all metabolites decrease in BALB/c mice independent of the cholecalciferol level in the diet. A similar tendency was observed in C57BL/6 mice. In addition, the lowest levels of all metabolites were observed in both strains of mice on the last day of observation after calcitriol administration to mice fed with the deficient diet (Figure 4B,C).

Analyzing the kinetics of the metabolite level, some differences were noted between the two mice strains; therefore, in the two measurement points, which were the same for both strains, we compared the metabolite levels between them. Interestingly, we observed that the levels of all metabolites were lower in C57BL/6 mice, even when they were fed with the control diet (day -7/week 5). After the next 3 weeks (day 14/week 8), the plasma levels of all metabolites were higher in C57BL/6 than in BALB/c mice (Figure 4G–I).

### 3.3. Vitamin D Metabolite Plasma Levels in Mammary Gland Tumor-Bearing Mice

**Figure 5:**
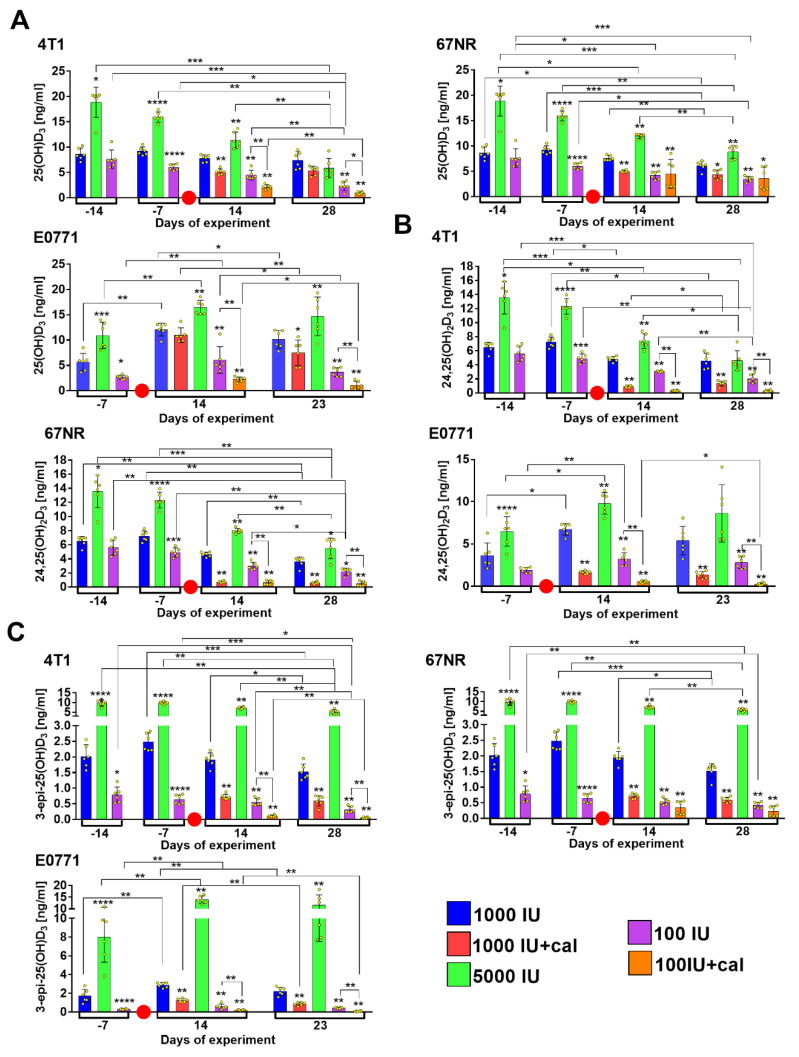
Kinetics of vitamin D metabolite plasma level in mammary gland tumor-bearing BALB/c and C57BL/6 mice. (**A**) 25(OH)D_3_, (**B**) 24,25(OH)_2_D_3_, and (**C**) 3-epi-25(OH)D_3_ plasma levels in mice bearing 4T1, 67NR (BALB/c), and E0771 (C57BL/6) mammary gland cancer. A red circle separates the measurements taken before the tumor implantation from those taken after the implantation. Data presented as mean with standard deviation; individual data are also presented as yellow points. Plasma from 6 mice/group was analyzed. Statistical analysis: Dunn’s test for multiple comparisons or Mann–Whitney test. * *p* < 0.05; ** *p* < 0.01; *** *p* < 0.001; **** *p* < 0.0001.

**Figure 6:**
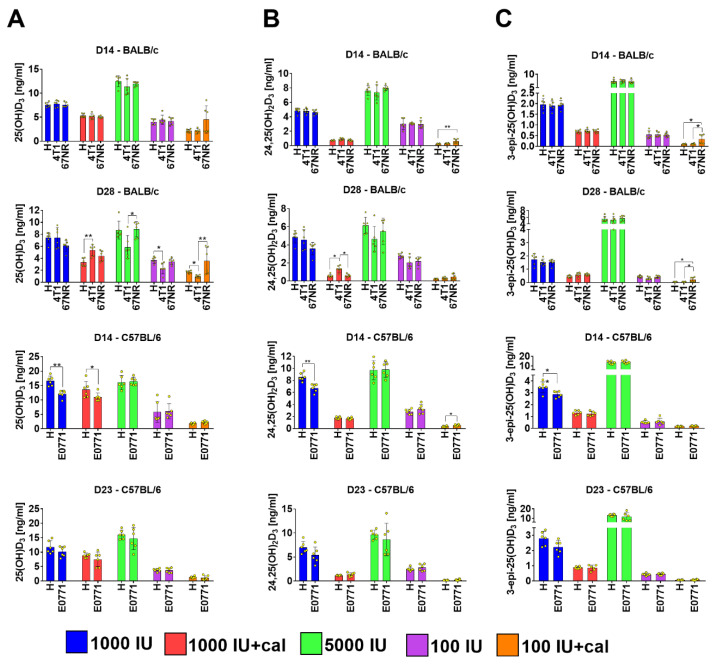
Comparison of vitamin D metabolite plasma level in healthy (H) and mammary gland tumor-bearing BALB/c and C57BL/6 mice. (**A**) 25(OH)D_3_, (**B**) 24,25(OH)_2_D_3_, and (**C**) 3-epi-25(OH)D_3_ plasma levels in mice bearing 4T1, 67NR (BALB/c), and E0771 (C57BL/6) mammary gland cancer. Data are presented as mean with standard deviation; individual data are also presented as yellow points. Plasma from 6 mice/group was analyzed. Statistical analysis: Dunn’s test for multiple comparisons or Mann–Whitney test. * *p* < 0.05; ** *p* < 0.01.

The general metabolite profiles did not change after tumor transplantation as compared to the profiles of healthy mice (Figure 5 vs. Figure 4). However, we observed some changes in the levels of particular metabolites between healthy and tumor-bearing mice or between the mice bearing tumors with different degrees of invasiveness (Figure 6). When we compared the plasma levels of all metabolites in control mice (1000 IU), we found only a decrease in levels in C57BL/6 mice bearing E0771 tumors (Figure 6A–C, day 14) as compared to healthy mice. In 4T1 tumor-bearing BALB/c mice fed with normal diet and injected with calcitriol, the plasma levels of 25(OH)D_3_ and 24,25(OH)_2_D_3_ were higher as compared to healthy mice, and the levels of 24,25(OH)_2_D_3_ were higher compared to 67NR tumor-bearing mice (Figure 6A,B, day 28). Mice fed with the diet supplemented with vitamin D had similar levels of all metabolites independent of the experimental condition. The exception was the plasma level of 25(OH)D_3_ which was significantly higher in the 67NR group as compared to 4T1 tumor-bearing mice (Figure 6A, day 28). Vitamin D deficiency led to the lowest 25(OH)D_3_ plasma level in mice bearing 4T1 cancer cells; a similar effect was observed in mice fed with the deficient diet and injected with calcitriol (Figure 6A, day 28). In this group of mice (100 IU+cal), we observed significantly higher plasma levels of 24,25(OH)_2_D_3_ (Figure 6B, day 14) and 3-epi-25(OH)D_3_ (Figure 6C, days 14 and 28) in 67NR tumor-bearing mice as compared to healthy mice.

### 3.4. Ratio of 25(OH)D_3_ to 24,25(OH)_2_D_3_ and to 3-epi-25(OH)D_3_

The ratio of 25(OH)D_3_:24,25(OH)_2_D_3_ significantly increased when BALB/c (Table 2) or C57BL/6 (Table 3), healthy or tumor-bearing mice were orally administered with calcitriol. This effect was seen in both mice receiving the standard and vitamin D-deficient diets as compared to the mice receiving the standard diet (1000 IU) alone. Additionally, a significant increase was observed in the deficient group (100 IU) and 100 IU+cal group (Table 2 and Table 3). In 4T1 and E0771 tumor-bearing mice administered calcitriol, the 25(OH)D_3_:24,25(OH)_2_D_3_ ratio was lower than in healthy mice (Table 2 and Table 3).

Vitamin D_3_ deficiency increased the 25(OH)D_3_:3-epi-25(OH)D_3_ ratio, while supplementation significantly decreased it (Table 2 and Table 3). However, calcitriol administration to vitamin D_3_-deficient mice led to especially high levels of this ratio. An increase (but not so high) was also observed in the 25(OH)D_3_:3-epi-25(OH)D_3_ ratio in mice receiving the normal diet and calcitriol as compared to the control group (Table 2 and Table 3). In 4T1 or 67NR tumor-bearing mice from the 100 IU+cal group, the calculated ratio was generally lower than that of healthy mice (Table 2).

### 3.5. Blood Biochemical Parameters

The plasma level of Ca^2+^ and phosphate did not change due to the diet fed and/or treatment in BALB/c healthy and 67NR tumor-bearing mice. The calcium level was increased only in 4T1 tumor-bearing BALB/c mice that were treated with calcitriol. However, in C57BL/6 mice, the plasma level of calcium was increased in both calcitriol-treated, healthy and E0771 tumor-bearing mice (Table 4). The phosphate level was the lowest in 4T1 and 67NR tumor-bearing mice that were on vitamin D-deficient diet and gavaged with calcitriol. In C57BL/6 mice, the phosphate level was increased in the group treated with calcitriol (in both mice fed with the normal and deficient diets). Similar to calcium levels, an increase in phosphate was observed in C57BL/6 mice fed with the diet containing 5000 IU of cholecalciferol. Among C57BL/6 mice, the differences in phosphate level were more pronounced in healthy mice (Table 4).

The diets and calcitriol treatment did not affect the level of creatinine. Only mice bearing 4T1 tumors had a higher plasma level of creatinine than healthy mice in the same treatment group. Moreover, in mice bearing 67NR tumors from the 100 IU+cal group, the creatinine level was higher than in 4T1 tumor-bearing mice from the corresponding group (Table 4). The level of alkaline phosphate also did not differ according to the diet or calcitriol treatment. However, both BALB/c and C57BL/6 mice bearing mammary gland tumors had a lower level of this enzyme as compared to healthy mice.

The total protein level was significantly increased in 4T1 tumor-bearing mice on the 100 IU diet and treated with calcitriol as compared to 4T1 mice fed with the diet containing a normal level of cholecalciferol. Among 4T1 tumor-bearing mice, high total protein levels (higher than in healthy BALB/c mice) were also observed in the 1000 IU+cal and 5000 IU groups. On the contrary, total protein levels were the lowest in C57BL/6 mice bearing E0771 cells in both calcitriol-treated and 5000 IU groups. The level of albumin did not change significantly among healthy BALB/c mice. In healthy C57BL/6 mice, a significantly increased level of albumin was observed in mice from 1000 IU+cal and 5000 IU groups, and the same tendency was observed in the 100 IU+cal group (*p* = 0.0729) as compared to the 1000 IU control group (Table 4). E0771 tumor-bearing mice had, in general, a lower level of albumin than healthy mice from the corresponding group (Table 4).

### 3.6. VDR, CYP2R1, CYP27B1, and CYP24A1 Expression in Tumor Tissue, Liver, and Kidney

**Figure 7:**
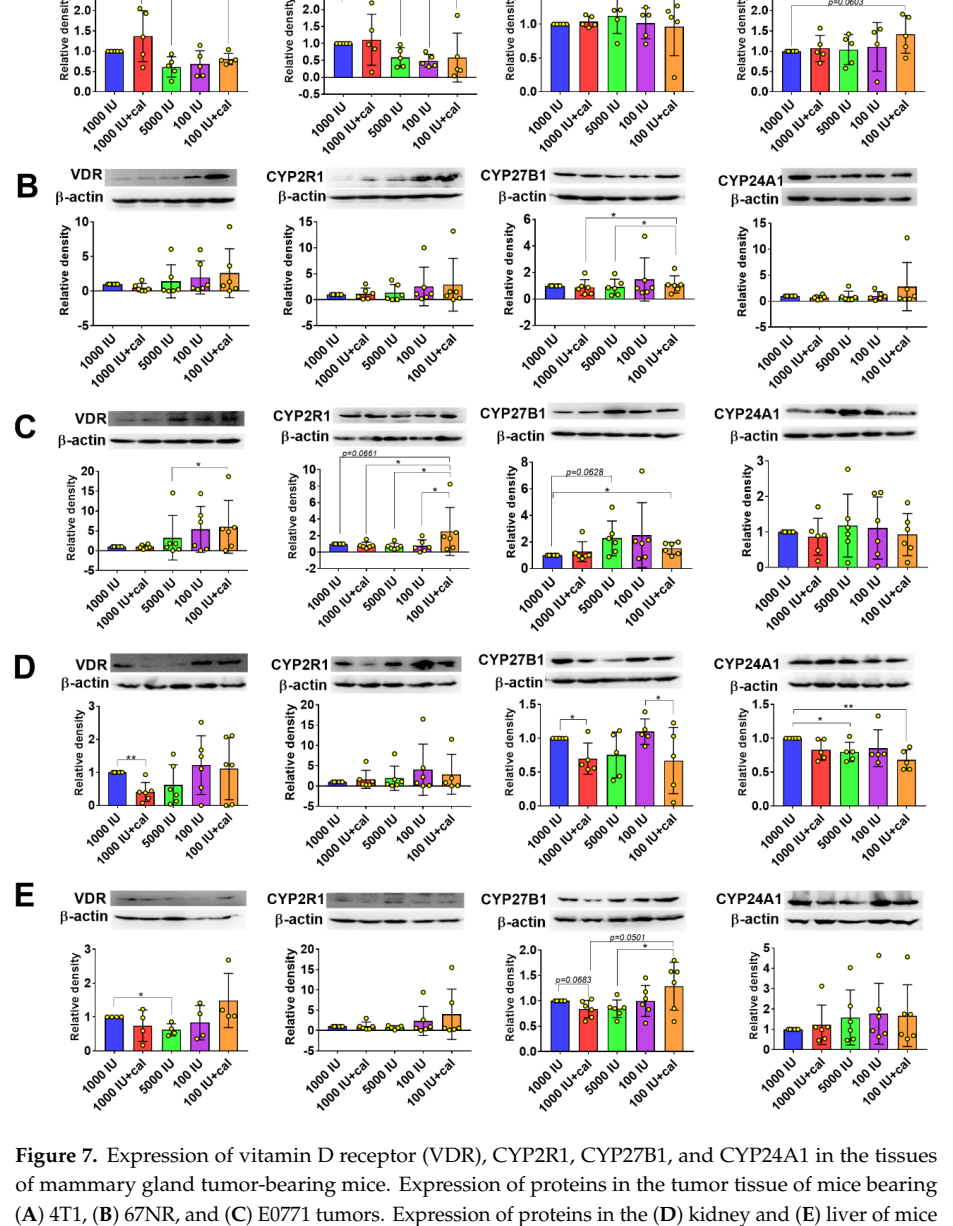
Expression of vitamin D receptor (VDR), CYP2R1, CYP27B1, and CYP24A1 in the tissues of mammary gland tumor-bearing mice. Expression of proteins in the tumor tissue of mice bearing (**A**) 4T1, (**B**) 67NR, and (**C**) E0771 tumors. Expression of proteins in the (**D**) kidney and (**E**) liver of mice bearing E0771 tumors. N = 4–6 mice per group. Densitometric analysis was performed using ImageJ software. Results are normalized to β-actin and next to the control group (1000 IU). Data are presented as mean with standard deviation; individual data are also presented as yellow points. Tissue from 5–6 mice/group was analyzed. Statistical analysis: Sidak’s test for multiple comparisons. * *p* < 0.05; ** *p* < 0.01.

The expression of VDR in 4T1 tumor tissue was lower among mice fed with the vitamin D-deficient diet and gavaged with calcitriol (100 IU+cal). A similar tendency was observed in mice fed with the 5000 and 100 IU diets (Figure 7A). In 67NR and E0771 tumors, we observed a similar trend (but opposite to 4T1); however, these results were not statistically significant (Figure 7B,C).

Nuclear expression of VDR was examined by immunohistochemistry in tumor tissue and in the lungs. The most intensive reaction (positive staining >50%) was observed in 4T1 tumor tissue (Suppl. Figure S1A), while a 25–50% staining was noticed in 67NR tumors (Suppl. Figure S1C) and the lowest staining (1–10%) in E0771 tumors (Suppl. Figure S1E). Only in 4T1 tumors from the 1000 IU+cal group was a significant decrease in VDR nuclear staining observed as compared to the 1000 IU group, whereas in E0771 tumor-bearing mice from the 100 IU+cal group increased VDR staining was found as compared to 100 IU (Suppl. Figure S1A,E). Analysis of lungs from healthy mice revealed no VDR staining (Suppl. Figure S1G, representative picture), whereas the highest lung staining was again found in 4T1 tumor-bearing mice (25–50%), followed by E0771 mice (0–10%), and the lowest staining in 67NR tumor-bearing mice (only in one mouse from the 5000 IU group) (Suppl. Figure S1B,D,F). Positive nuclear staining was observed preferentially in tumor cells.

CYP2R1 expression in 4T1 tumor tissue was the lowest in mice fed with the 5000 and 100 IU diets (Figure 7A). Its expression did not change significantly by the treatments in 67NR tumors (Figure 7B). A significantly higher expression of CYP2R1 was noticed in E0771 tumors from the 100 IU+cal group as compared to all other groups (Figure 7C). Similarly, the expression of CYP27B1 was changed only in E0771 tumors; for instance, a significantly increased level was found in mice fed with 100 IU+cal (Figure 7C). A similar tendency was observed in mice from the 5000 IU group (*p* = 0.0628; Figure 7C). The highest expression of CYP24A1 was observed in 4T1 tumors from mice treated with the 100 IU diet and calcitriol (*p* = 0.0603; Figure 7A).

The expression of these proteins was also studied in the kidney and liver of E0771 tumor-bearing mice. The pattern of VDR expression (dependent on the treatment) seemed to be similar in E0771 tumors and kidneys (Figure 7C,D, respectively); however, in the kidneys, the decrease in VDR expression was statistically significant in mice from the 1000 IU+cal group (Figure 7D). The expression of CYP2R1 did not change significantly, but CYP27B1 expression was lowered after gavage with calcitriol in mice fed with the 1000 and 100 IU diets (1000 IU+cal as compared to 1000 IU and 100 IU+cal as compared to 100 IU; Figure 7D). Moreover, CYP24A1 expression was decreased in mice from the 5000 IU and 100 IU+cal groups as compared to the 1000 IU group (Figure 7D). In the liver, among the proteins tested, only VDR expression was lowered in mice fed with the vitamin D-supplemented diet (5000 IU; Figure 7E). The opposite tendency was observed among mice treated with calcitriol for the expression of CYP27B1—it was lower in the 1000 IU+cal group and higher in the 100 IU+cal group (Figure 7E).

**Figure 8:**
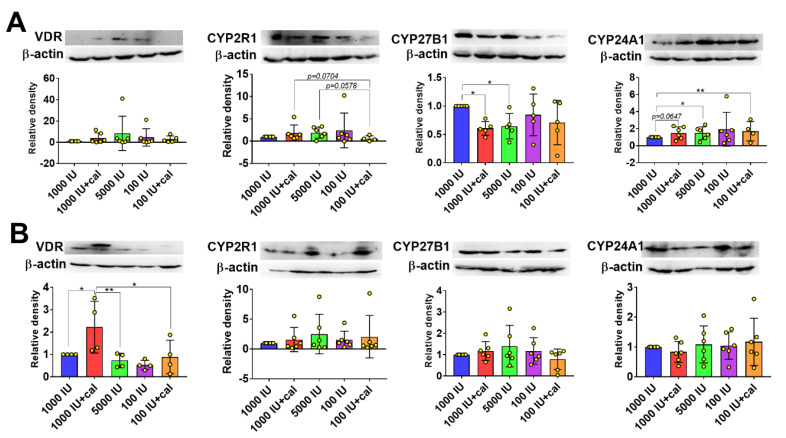
Expression of VDR, CYP2R1, CYP27B1, and CYP24A1 in the tissues of healthy C57BL/6 mice. Expression of proteins in the (**A**) kidney and **(B**) liver. N = 4–6 mice per group. Densitometric analysis was performed using ImageJ software. Results are normalized to β-actin and next to the control group (1000 IU). Data are presented as mean with standard deviation; individual data are also presented as yellow points. Tissue from 4–6 mice/group was analyzed. Statistical analysis: Sidak’s test for multiple comparisons. * *p* < 0.05; ** *p* < 0.01.

In the case of healthy C57BL/6 female mice, upon various treatments, VDR expression in the kidney did not change (Figure 8A), but the expression was significantly increased in the liver in the 1000 IU+cal group (Figure 8B). Among healthy C57BL/6 mice, CYP2R1 expression in the kidney was the lowest in the 100 IU+cal group (Figure 8A). The expression of CYP27B1 in kidneys was lowered in all treatment groups of healthy mice as compared to control (1000 IU), but statistically significant differences were noticed only in two groups: 1000 IU+cal and 5000 IU (Figure 8A). CYP24A1 expression in the kidney was increased in all treatment groups of healthy C57BL/6 mice, but a significant increase was observed only in the 5000 IU and 100 IU+cal groups with significant tendency in the 1000 IU+cal group (*p* = 0.0647; Figure 8A). The expression of vitamin D-metabolizing enzymes was not changed significantly in the liver of healthy C57BL/6 mice upon treatments used (Figure 8B).

### 3.7. Blood Morphological Parameters Measured on the Last Day of Observation

In healthy mice, diet modifications or calcitriol gavage did not significantly change most populations of white blood cells (WBCs). Only the general number of WBCs was decreased in BALB/c mice on the vitamin D-deficient diet, while in C57BL/6 mice it was increased in the 1000 IU+cal group as compared to the control (1000 IU) healthy mice (Table 5). The progression of 4T1 is accompanied by leukocytosis. In our study, vitamin D deficiency did not significantly affect the number of WBC populations; however, the highest numbers of WBCs and lymphocytes, granulocytes, and monocytes were observed in 4T1-bearing mice on the vitamin D-deficient diet supplemented with calcitriol (100 IU+cal group). In mice bearing 67NR tumors, the WBC number did not change according to the treatment groups. However, mice bearing E0771 tumors had increased numbers of WBC, monocytes, and granulocytes when treated with calcitriol (1000 IU+cal) compared to healthy mice treated in the same way. Mice bearing E0771 tumors and fed vitamin D-deficient food (with or without calcitriol gavage) showed increased numbers of WBCs and especially lymphocytes (Table 5).

The morphology of erythrocytes and platelets is presented in Suppl. Figures S2–S4. The erythrocyte number was lower in mice bearing 4T1 cells and fed the vitamin D-deficient diet supplemented or not with calcitriol gavage as compared to tumor-bearing mice fed with the normal diet. Additionally, in the 100 IU+cal group with 4T1 tumors, the mean cell volume and mean corpuscular hemoglobin were significantly increased (Suppl. Figures S2 and S3). E0771 tumor-bearing mice on a normal diet gavaged with calcitriol (1000 IU+cal) had decreased hematocrit and hemoglobin as well as increased mean platelet volume with the lowest number of platelets as compared to control tumor-bearing mice (1000 IU) (Suppl. Figure S4).

## 4. Discussion

**Figure 9:**
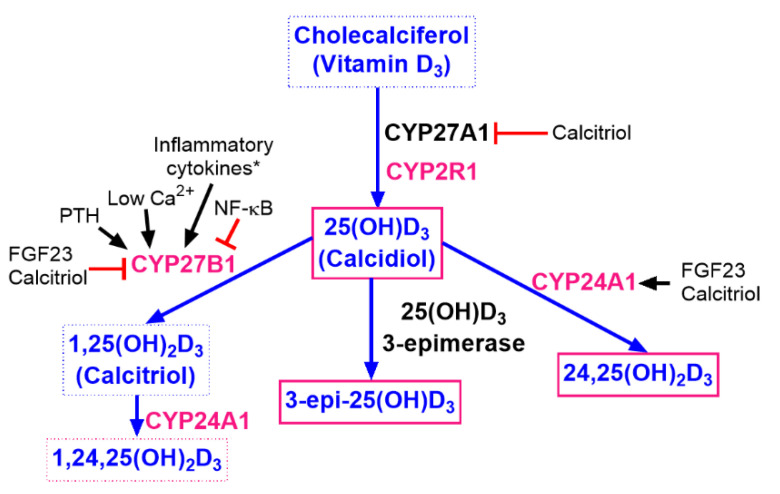
Summary of vitamin D_3_ metabolic pathways with selected factors playing an important role in their regulation. CYP27A1 and CYP2R1 are identified as vitamin D_3_-25-hydroxylases converting cholecalciferol to 25(OH)D_3_. CYP27B1: 25-hydroxyvitamin D-1α-hydroxylase converts 25(OH)D_3_ to its hormonally active form—1,25(OH)_2_D_3_, calcitriol. CYP24A1: 24-hydroxylase inactivating both 25(OH)D_3_ and 1,25(OH2)D_3_. Regulation of CYPs’ expression may differ depending on the tissue studied. * Inflammatory cytokines such as IFNγ, IL-1β, IL-15, IGF-I, EGF, and TGF-β activate CYP27B1 [34]. Pink letters: enzymes measured in our study in the kidney, liver, and tumor tissue. Pink frames: metabolites measured in mice plasma in our study.

The varied effects of vitamin D supply in the form of cholecalciferol or calcitriol on the metabolite profile of vitamin D_3_ result from the fact that both substances act as: 1) a “substrate” in the initial stages of metabolism and 2) the “final molecule”—the active form, respectively, and differently affect the vitamin D metabolic machinery (the schematic presentation of vitamin D metabolism, starting from cholecalciferol, is presented in Figure 9).

Therefore, we observed an upregulation of all the tested metabolites when the mice were fed with diet enriched with 5000 IU of cholecalciferol and a decrease in these levels when calcitriol was administered. A similar tendency was observed in the study of Halloran et al. conducted on rats [35] and in the studies on humans [36,37]. In a study on rats that received chronic injections of calcitriol, the authors concluded that “chronic 1,25(OH)_2_D_3_ administration lowers serum 25(OH)D_3_ by increasing the metabolic clearance of 25(OH)D_3_ and not by decreasing its production” [35]. In normal conditions, renal CYP27B1 determines the circulating supply of calcitriol and its expression is regulated by many factors related to calcium homeostasis, including calcitriol, which downregulates CYP27B1 [34]. A decrease in the level of CYP27B1 in the kidney was observed in our study, mainly in the treatment groups of healthy C57BL/6 mice receiving calcitriol and the 5000 IU diet, as compared to the mice fed with a normal diet. A similar tendency was observed in the kidney of C57BL/6 tumor-bearing mice treated with calcitriol. This effect correlated with the increase in plasma Ca^2+^ in mice gavaged with calcitriol, but not in the 5000 IU group. However, in our study, we observed that calcitriol administration decreased the activity of CYP24A1 (but not in mice fed with the cholecalciferol-supplemented diet—5000 IU), which was measured as the increased ratio of plasma 25(OH)D_3_:24,25(OH)_2_D_3_. Scientific data suggest that a relative decrease in the plasma level of 24,25(OH)_2_D_3_ with a decreased level of 25(OH)D_3_ indicates the downregulation of CYP24A1 activity. Moreover, low serum levels of 24,25(OH)_2_D_3_ and the elevated 25(OH)D_3_:24,25(OH)_2_D_3_ ratio are useful in identifying patients with loss-of-function *CYP24A1* mutations [38]. On the other hand, we observed an increased expression of CYP24A1 protein in the kidney of healthy mice treated with calcitriol, as well as in the mice fed with the vitamin D_3_-supplemented diet. This negative feedback mechanism, which increases the kidney expression of CYP24A1 enzyme that degrades vitamin D and all its metabolites, is known as calcitriol attribute [39]. However, in C57BL/6 mice, CYP24A1 kidney expression in the 5000 IU and 100 IU+cal groups (with a similar tendency in 1000 IU+cal) differed between healthy and tumor-bearing C57BL/6 mice—CYP24A1 was downregulated in tumor-bearing and upregulated in healthy mice. However, in both tumor-bearing and healthy mice, the 25(OH)D_3_:24,25(OH)_2_D_3_ ratio was highly increased in the 100 IU+cal and 1000 IU+cal groups and not changed or slightly decreased in the 5000 IU group, independent of the level of CYP24A1 protein. This indicates the decrease in enzyme activity and its tendency to increase [38], respectively, independent of tumor burden.

In C57BL/6 mice bearing E0771 tumors which were fed with the vitamin D-deficient diet and injected with calcitriol (100 IU+cal), we observed an increase in the level of CYP27B1 with an increase in CYP2R1 in tumor tissue. In tumor-bearing BALB/c mice, such an effect was not observed. However, the level of CYP24A1 was the highest in 4T1 tumor tissue from the same treatment group (100 IU+cal), and with the low level of CYP2R1 protein in these mice we observed the lowest plasma level of 25(OH)D_3_. Although CYP2R1 and CYP27A1 are identified as vitamin D_3_-25-hydroxylases converting cholecalciferol to 25(OH)D_3_, studies have shown that, for example, deletion of their genes in mice did not result in complete lack of plasma 25(OH)D_3_, which suggests the possibility that several hydroxylases could contribute to the conversion process [34]. In our study, we only focused on CYP2R1 expression, because this enzyme is described as a more important 25-hydroxylase, the deletion of which might lead to a significant decrease in the level of 25(OH)D_3_ in mice [40]. Our observations in the tumor tissue of metastatic breast cancers 4T1 and E0771, especially in mice fed with the vitamin D-deficient diet and simultaneously treated with calcitriol, showed the impact of such a treatment on the expression of this enzyme. However, additional studies are needed to explore the mechanisms and the importance of this phenomenon. There is evidence that BALB/c and C57BL/6 mice differ in the susceptibility to mammary tumors [41]. Genomic studies identify the *Cyp2r1* gene as located in the so-called *SuperMam1* locus, which is a mammary tumor susceptibility locus in the BALB/cTrp53+/-strain (model of spontaneous breast cancer) [42,43]. Further studies documented that *Cyp2r1* mRNA levels were significantly lower in the mammary glands of BALB/c mice (and in C57BL/6 mice with the *SuperMam1* locus) compared to that in the wild-type C57BL/6 mice, suggesting that the local dysregulation of *Cyp2r1* may contribute to the development of mammary cancer by decreasing the local supply of vitamin D [44]. Therefore, the decrease in the level of CYP2R1 observed in 4T1 tumor tissue may be responsible for the increased invasive potential of these cells. In addition, evidence shows the impact of CYP27B1 on the development of breast cancer. For instance, specific knockout of the *Cyp27b1* gene in mammary epithelium contributed to the accelerated growth of mammary tumor probably as a result of decreased local synthesis of calcitriol [45]. On the other hand, in the mouse model of thyroid cancer, *Cyp24a1* knockout led to a decline in tumorigenesis, which directly proved that *Cyp24a1* functions as an oncogene [46].

**Figure 10:**
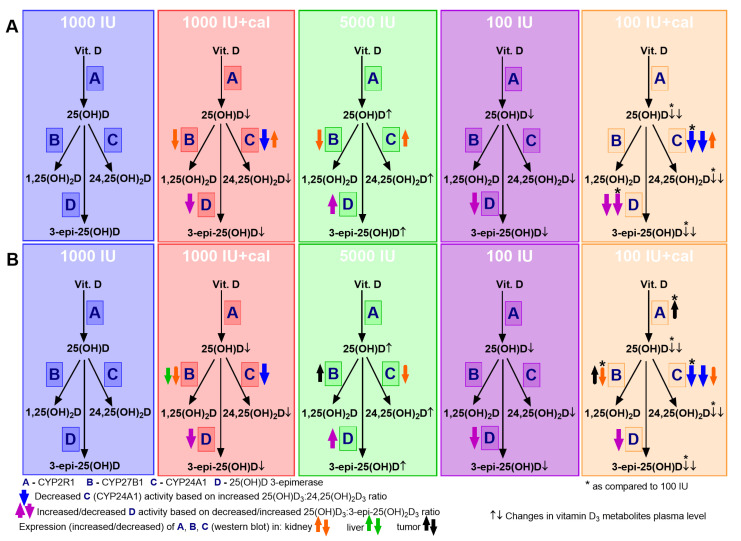
Summary of the elements of vitamin D metabolism studied in mice in various experimental conditions. All changes are indicated as compared to control (1000 IU) with the exception of those marked with *, which are compared to 100 IU (**A**) healthy; (**B**) E0771 tumor-bearing C57BL/6 mice. The general idea of this scheme comes from the paper of Schoenmakers et al. [53].

Recently conducted research has shown the importance of the 3-epimerization process during the metabolism of vitamin D (reviewed in detail elsewhere [47]). 3-Epimerase catalyzes the 3β-to-3α epimerization of 25(OH)D_3_ resulting in 3-epi-25(OH)D_3_, which is one of the most abundant metabolites of vitamin D in the serum and can be converted to 3-epi-1α,25(OH)_2_D_3_ by CYP27B1 [48]. All major vitamin D metabolites can be epimerized at the C3 position. Docking studies suggest that 3-epi-1α,25(OH)_2_D_3_, unlike calcitriol, binds to CYP24A1 in an alternate configuration. Such a configuration destabilizes the enzyme–substrate complex and slows the rate of inactivation of 3-epi-1α,25(OH)_2_D_3_ by CYP24A1 by metabolizing it into 3-epi-calcitroic acid [49]. A study also showed that 3-epi-1,25(OH)_2_D_3_ stimulates the transcription of genes acting through VDR, despite the fact that its affinity to VDR is lower than calcitriol [50]. The high biological activity of 3-epi metabolites is described as an effect of its high stability [51]. It is suggested that the 3-epimerization process may lead to the overestimation of vitamin D status, and in mice (but not in humans) it was noticed that oral vitamin D supplementation leads to an increased production of epimers [52]. Similarly, in our study, mice fed with the diet containing 5000 IU of cholecalciferol had the highest levels of 3-epi-25(OH)D_3_ compared to all other treatment groups. Moreover, the 25(OH)D_3_:3-epi-25(OH)D_3_ ratio was the lowest in these mice, suggesting increased 3-epimerase activity (summarized in Figure 10A). Except for the groups fed with the high-vitamin D_3_ diet, calcitriol was administered to mice fed with a diet containing normal content of vitamin D_3_—i.e., 1000 IU, and mice fed with vitamin D_3_-deficient diet. These protocols, and especially the last one, led to the lowest levels of all metabolites measured. The last protocol also resulted in the highest increase in the 25(OH)D_3_:3-epi-25(OH)D_3_ ratio, probably due to a significant decrease in 3-epimerase activity in this experimental condition. Similarly, a high 25(OH)D_3_:3-epi-25(OH)D_3_ ratio was observed in the second group of mice treated with calcitriol (1000 IU+cal) as well as in the mice fed with the vitamin D-deficient diet (100 IU). This suggests that both vitamin D_3_ deficiency and calcitriol supply, especially in the case of vitamin D deficiency, cause a reduction in vitamin D metabolites circulating in the blood, including 3-epi metabolites, while the opposite effect was observed in the mice fed with the cholecalciferol-enriched diet (Figure 10).

The above observations were similar in both strains of mice (BALB/c and C57BL/6), but some differences were observed in the time-kinetics of the metabolites level. In BALB/c mice, a time-dependent decrease was noted in the level of all metabolites in all (including healthy) groups of mice. However, such a decrease was not observed in C57BL/6 mice. This difference in the time-related kinetics was the reason for higher plasma levels of metabolites in BALB/c mice in the beginning of the experiment and the lowest level at the end as compared to C57BL/6. Strain-dependent differences in the expression of CYP2R1 described above may contribute to the different time-related profiles of metabolites between the two strains [44].

The metabolic profiles observed in our study in healthy mice were, in general, convergent in tumor-bearing mice (with exceptions described above, Figure 10); however, diets containing various levels of vitamin D_3_ and calcitriol had different effects on the body weight of tumor-bearing mice as compared to healthy mice. The body weight of healthy BALB/c and C57BL/6 mice was stable during the experiment, whereas a significant reduction in body weight was observed in 4T1 tumor-bearing BALB/c mice administered calcitriol (and fed with the normal and deficient diets). In addition, 67NR and E0771 tumor-bearing mice from the same treatment groups showed a significant decrease in body weight, but this decrease started at the end of the observation period. This effect was accompanied by an increase in calcium level (in 4T1 and E0771 mice). However, the highest changes of blood biochemical parameters were observed in 4T1 tumor-bearing mice from the 100 IU+cal group, which had decreased phosphate and albumin levels and increased total protein in the plasma. Progression of 4T1 tumors is accompanied by a large inflammatory response [54]. In our previous studies, in which 4T1 tumor-bearing mice were injected subcutaneously with calcitriol, the inflammatory response increased as compared to 4T1 control mice [55,56]; however, in the current study, the intensity of the WBC count increase was the highest in the mice fed with the vitamin D-deficient diet and gavaged with calcitriol (100 IU+cal), in which the lowest plasma levels of all vitamin D_3_ metabolites were also observed. Such intensification was not observed in 67NR, nonmetastatic (isogenic to 4T1) cell line-bearing mice. Interestingly, calcitriol (as well as vitamin D deficiency) also led to an increase in WBC count in E0771 tumor-bearing mice, but the progression of this tumor did not induce any inflammatory response [31].

The above data show that the diet level of vitamin D, as well as the exogenously supplied calcitriol, has different effects on the body condition, depending on the burden of breast cancer, and more importantly, the type of breast cancer. It reflects as increased metastatic potential of 4T1 mammary gland cancer in calcitriol-treated mice, as well as in mice fed with vitamin D_3_-supplemented (5000 IU) or vitamin D_3_-deficient (100 IU) diets, despite the differences in vitamin D metabolite profiles and vitamin D status (measured as the plasma level of 25(OH)D_3_) at the day of tumor inoculation. In E0771 tumor-bearing mice fed with the vitamin D-deficient diet, we observed an increased number of metastases (not statistically significant), which was not attenuated by calcitriol gavage. Interestingly, mice bearing nonmetastatic 67NR cells fed with vitamin D_3_-supplemented and vitamin D_3_-deficient diets were also diagnosed with single metastatic foci in the lungs. Therefore, our data suggest that in some types of mammary gland cancer, an excessive or insufficient vitamin D supply may lead to unfavorable effects, namely increased tendency of metastasis. It was previously demonstrated that vitamin D deficiency promoted the metastasis of human breast cancer cells in the experimental model of bone metastasis in nude mice [15]. In the model of MMTV-PyMT spontaneous mouse mammary gland cancer, an injection of 25(OH)D delayed not only tumor growth but also metastasis [57]. These two models of breast cancer [15,57], similar to 67NR [22] cells, are sensitive to in vitro vitamin D treatment contrary to 4T1 [22] and E0771 (Suppl. Figure S5).

Other data have shown that calcitriol or its analogs may inhibit the primary tumor growth of breast cancer xenografts [27,29] or transplantable mouse mammary gland cancer [19,28,58]. Moreover, improved mammary gland tumor (MMTV-wt) growth was observed in mice with vitamin D deficiencies [16]. On the other hand, stimulation of primary 4T1 tumor growth was caused by vitamin D_3_ in another study [24]. Our previous [22] and current results did not show any effect of various vitamin D statuses on 4T1 primary tumor growth; however, we observed a transient inhibition of 67NR tumor burden by calcitriol in mice fed with the normal diet (1000 IU+cal). A similar trend (without statistical significance) was noticed in E0771 tumors from the same treatment group and from the group fed with the 5000 IU diet. Recently, Karkeni et al. showed that E0771 tumor growth can be inhibited by cholecalciferol gavage in normal C57BL/6 mice, but in mice fed with the high-fat diet, the effect of vitamin D was opposite [59]. This is a very interesting observation, based on which the authors concluded that the inhibition of primary tumor growth by vitamin D_3_ is correlated with the tumor infiltration of CD8^+^ T lymphocytes. Moreover, the authors showed a decrease in the infiltration of proinflammatory macrophages in peripheral tissues and not in the tumor [59]. Our previous studies in a 4T1 mammary gland cancer model have shown that modulation of the tumor microenvironment by calcitriol or its analogs plays a fundamental role in controlling the progression of these cancer cells [30,55,56,60]. An inflammatory process often accompanies cancer progression and actively contributes to the survival of cancer cells, angiogenesis, and metastasis [61]. It is known that, for example, tumor-associated fibroblasts and tumor cells build an inflammatory milieu that is favorable for the recruitment of Th17 cells [62]. We have observed differences in immune response of these pro-inflammatory lymphocytes, depending on the age of 4T1 tumor-bearing mice after treatment with a calcitriol analog (tacalcitol) [56]. This included increased activity of cells in 4T1 young tumor-bearing mice [56] in which increased metastatic potential [22] and body response toward Th2 and Treg cells with a decrease in NK and CD8^+^ T cells were also observed [55]. In aged ovariectomized mice, transient antimetastatic effects of calcitriol or its analogs were observed with decreased activity of Th17 cells and decreased Treg cells [30,55,56]. Therefore, current and previous studies suggest that the modulation of tumor growth and metastasis by vitamin D may be dependent not only on the type or characteristics of cancer cells but also on the body condition, including age and obesity. Epidemiological data which often did not lead to clear conclusions also support the data from animal experiments. Some examples include the study by Kanstrup et al. showing poorer breast cancer survival among women with high 25(OH)D levels [14] or a European population-based cohort study showing increased breast cancer risk with higher 25(OH)D concentrations among older adults [13].

## 5. Conclusions

Although varied effects were observed for supplementation (in the form of cholecalciferol or calcitriol) or deficiency of vitamin D on tumor growth and metastasis of different types of mammary gland cancer, metabolite profiles were similar in the plasma of healthy and tumor-bearing mice. On the other hand, the levels of vitamin D-metabolizing enzymes differed between kidney and tumor tissue in the studied mice, which suggests that the tumor microenvironment influences the final effects of vitamin D on tumor growth and metastasis.

## Figures and Tables

**Table 1:** Summary of experiments performed on animals.

Tumor Model/Mouse Strain/Cell Number	4T1—BALB/c 1 × 10^4^	67NR—BALB/c 2 × 10^5^	E0771—C57BL/6 5 × 10^4^
Autopsy day *	28	28	23
Diet cholecalciferol (vitamin D_3_) content; calcitriol p.o. injections	Control 1000 IU		
	Control 1000 IU+calcitriol from day 7 after tumor implantation (1 µg/kg/dose 3× a week)		
	Supplemented 5000 IU		
	Deficient 100 IU		
	Deficient 100 IU+calcitriol from day 7 after tumor implantation (1 µg/kg/dose 3× a week)		

* Healthy mice of both strains: BALB/c and C57BL/6, treated in the same way, were euthanized at the same time as the tumor-bearing mice, and the tissues were collected.

**Table 2:** Ratio of 25(OH)D_3_ to 24,25(OH)_2_D_3_ and to 3-epi-25(OH)D_3_ in BALB/c mice.

Time of Blood Collection	Group	BALB/c					
		25(OH)D_3_:24,25(OH)_2_D_3_			25(OH)D_3_:3-epi-25(OH)D_3_		
		Healthy	4T1	67NR	healthy	4T1	67NR
Day-14 (4 weeks)	1000 IU	1.3 ± 0.2			4.3 ± 0.6		
	5000 IU	1.4 ± 0.1			1.9 ± 0.2 *		
	100 IU	1.4 ± 0.2			10.2 ± 2.3 *		
Day-7 (5 weeks)	1000 IU	1.3 ± 0.1			3.7 ± 0.2		
	5000 IU	1.3 ± 0.1			1.6 ± 0.7 *		
	100 IU	1.2 ± 0.1			9.8 ± 2.2 *		
Day 14 (8 weeks)	1000 IU	1.6 ± 0.1 ^#^	1.6 ± 0.1 ^#^	1.7 ± 0.2	3.9 ± 0.4	3.6 ± 1.1	3.9 ± 0.5
	1000 IU+cal	7.3 ± 0.8 *	5.8 ± 0.7 *	7.0 ± 0.9 *	7.8 ±0.6 *	7.2 ± 0.4 *	7.1 ± 0.6 *
	5000 IU	1.6 ± 0.1 ^#^	1.5 ± 0.1 ^#^	1.5 ± 0.1	1.7 ± 0.2 *,^$^	1.5 ± 0.1 *	1.6 ± 0.2 *
	100 IU	1.2 ± 0.2	1.5 ± 0.3	1.4 ± 0.3	7.9 ± 1.5 *	8.2 ± 1.4 *	8.1 ± 2.0 *
	100 IU+cal	8.2 ± 0.5 *^,@^	6.5 ± 0.8 *^,@^	6.9 ± 1.3 *^,@^	20.0 ± 3.1 *^,@^	18.6 ± 3.1 *^,@^	13.8 ± 2.6 *^,@^
Day 28 (10 weeks)	1000 IU	1.5 ± 0.1	1.6 ± 0.1 ^$,#^	1.7 ± 0.2 ^$,#^	4.4 ± 1.0	4.9 ± 0.8 ^#,&^	4.0 ± 0.2
	1000 IU+cal	6.1 ± 0.9 *	4.2 ± 1.0 *^,&^	7.8 ± 2.0 *	7.6 ± 0.5 *	9.4 ± 2.2 *^,&^	7.4 ± 1.3 *
	5000 IU	1.4 ± 0.1 ^&^	1.3 ± 0.2 ^&^	1.7 ± 0.3 ^#^	1.6 ± 0.1 *	1.1 ± 0.2 *^,$,&^	1.6 ± 0.1 *^,$^
	100 IU	1.3 ± 0.2	1.1 ± 0.3	1.6 ± 0.2 ^#^	8.7 ± 2.5 *	7.5 ± 2.4	8.4 ± 1.4 *
	100 IU+cal	7.5 ± 1.3 *^,@^	3.6 ± 1.7 *^,@,&^	8.3 ± 2.4 *^,@^	32.6 ± 5.9 *^,@,&^	19.2 ± 8.3 *^,@^	17.9 ± 3.9 *^,@^

The ratio was calculated for each mouse. Plasma from 6 mice/group was analyzed. Statistical analysis: Dunn’s test for multiple comparisons or Mann–Whitney test. Data underlined: *p* < 0.05 as compared to healthy mice from the same treatment group. * *p* < 0.05 as compared to 1000 IU on the same day of analysis; ^$^ *p* < 0.05 as compared to day -14; ^#^ *p* < 0.05 as compared to day -7; ^&^ *p* < 0.05 as compared to day 14; ^@^ *p* < 0.05 as compared to 100 IU on the same day of analysis.

**Table 3:** Ratio of 25(OH)D_3_ to 24,25(OH)_2_D_3_ and to 3-epi-25(OH)D_3_ in C57BL/6 mice.

Time of Blood Collection	Group	C57BL/6			
		25(OH)D_3_/24,25(OH)_2_D_3_		25(OH)D_3_/3-epi-25(OH)D_3_	
		Healthy	E0771	Healthy	E0771
Day-7 (5 weeks)	1000 IU	1.6 ± 0.3		3.6 ± 1.6	
	5000 IU	1.7 ± 0.3		1.4 ± 0.3 *	
	100 IU	1.4 ± 0.3		8.7 ± 1.2 *	
Day 14 (8 weeks)	1000 IU	1.9 ± 0.2 ^#^	1.8 ± 0.2	4.9 ± 0.9 ^#^	4.2 ± 0.7
	1000 IU+cal	7.8 ± 1.7 *	6.7 ± 1.1 *	10.2 ± 1.0 *	9.1 ± 2.0 *
	5000 IU	1.7 ± 0.2	1.7 ± 0.1	1.2 ± 0.1 *	1.2 ± 0.1 *
	100 IU	2.0 ± 1.1	1.9 ± 0.4 ^#^	11.1 ± 6.5 *	11.2 ± 4.3 *
	100 IU+cal	5.4 ± 1.1 *^,@^	4.4 ± 1.4 *^,@^	10.6 ± 1.6 *^,&^	11.0 ± 2.2 *
Day 23 (9 weeks)	1000 IU	1.7 ± 0.4	2.0 ± 0.5	4.3 ± 1.0	4.6 ± 0.9
	1000 IU+cal	7.4 ± 0.6 *	5.6 ± 1.2 *	10.1 ± 0.5 *	8.6 ± 2.1 *
	5000 IU	1.7 ± 0.1	1.8 ± 0.3	1.2 ± 0.1 *	1.3 ± 0.2 *
	100 IU	1.5 ± 0.1	1.3 ± 0.1 ^&^	9.0 ± 1.0 *	8.1 ± 0.9 *
	100 IU+cal	6.8 ± 1.0 *^,@,&^	4.0 ± 1.6 *^,@,&^	21.2 ± 7.1 *^,@^	13.6 ± 5.8 *

The ratio was calculated for each mouse. Plasma from 6 mice/group was analyzed. Statistical analysis: Dunn’s test for multiple comparisons or Mann–Whitney test. Data underlined: *p* < 0.05 as compared to healthy mice from the same treatment group. * *p* < 0.05 as compared to 1000 IU on the same day of analysis; ^#^ *p* < 0.05 as compared to day -7; ^&^ *p* < 0.05 as compared to day 14; ^@^ *p* < 0.05 as compared to 100 IU on the same day of analysis.

**Table 4:** Plasma level of selected biochemical parameters of blood.

Parameter Measured	Group	BALB/c Mice			C57BL/6 Mice	
		Healthy	4T1	67NR	Healthy	E0771
Ca^2+^ (mmol/L)	1000 IU	3.1 ± 0.1	3.2 ± 0.2	3.2 ± 0.2	3.1 ± 0.2	3.2 ± 0.1
	1000 IU+cal	3.1 ± 0.2	3.4 ± 0.2 ^#^	3.3 ± 0.2	3.4 ± 0.2 *	3.6 ± 0.2 *
	5000 IU	3.1 ± 0.1	3.3 ± 0.2	3.2 ± 0.2	3.2 ± 0.2	3.2 ± 0.2
	100 IU	3.1 ± 0.1	3.2 ± 0.2	3.2 ± 0.2	3.1 ± 0.1	3.1 ± 0.2
	100 IU+cal	3.4 ± 0.2	3.4 ± 0.3 *	3.3 ± 0.3	3.4 ± 0.2 *^,@^	3.6 ± 0.3 ^#,^*^,@^
Phosphate (mmol/L)	1000 IU	2.1 ± 0.4	2.6 ± 0.9	2.1 ± 0.5	2.4 ± 0.6	2.2 ± 0.7
	1000 IU+cal	2.0 ± 0.4	2.1 ± 0.5	2.0 ± 0.5	3.4 ± 0.7 *	2.3 ± 0.6 ^#^
	5000 IU	2.0 ± 0.4	2.6 ± 0.7	2.3 ± 0.4	3.3 ± 0.3 *	2.8 ± 0.6 *
	100 IU	2.1 ± 0.4	2.7 ± 0.5	2.2 ± 0.8	2.7 ± 0.4	2.5 ± 0.7
	100 IU+cal	2.4 ± 0.9	1.6 ± 0.3 ^#,^*^,@^	1.5 ± 0.3 ^#^	3.3 ± 0.3 *^,@^	2.4 ± 0.6 ^#^
Creatinine (µmol/L)	1000 IU	13.4 ± 1.6	9.6 ± 1.0 ^#^	11.4 ± 1.8	9.6 ± 2.6	11 ± 2.5
	1000 IU+cal	12.6 ± 3.0	9.6 ± 2.6 ^#^	11.3 ± 0.8	8.6 ± 2.2	9.4 ± 1.5
	5000 IU	12.8 ± 2.0	8.6 ± 2.6 ^#^	10.6 ± 2.5	10.5 ± 2.1	9.5 ± 2.4
	100 IU	12.9 ± 2.5	11.6 ± 4.5	10.5 ± 1.3	8.8 ± 1.5	8.9 ± 1.0
	100 IU+cal	12.2 ± 0.3	9.5 ± 1.0 ^#^	11.5 ± 1.6 ^&^	9.8 ± 0.9	9.2 ± 1.2
Alkaline phosphatase (U/L)	1000 IU	67.0 ± 10.0	39.1 ± 6.0 ^#^	49.3 ± 10.4 ^#^	72.8 ± 25.4	39.8 ± 23.9 ^#^
	1000 IU+cal	69.0 ± 5.8	36.6 ± 4.5 ^#^	49.3 ± 14.5 ^#^	55.1 ± 4.4	29.5 ± 17.8
	5000 IU	66.5 ± 6.6	38.4 ± 4.8 ^#^	45.1 ± 6.5 ^#^	76.4 ± 11.6	41.1 ± 20.2 ^#^
	100 IU	65.1 ± 6.1	38.2 ± 2.4 ^#^	50.4 ± 7.2 ^#^	82.4 ± 13.5	40.9 ± 21.2 ^#^
	100 IU+cal	67.9 ± 5.3	34.2 ± 4.0 ^#^	43.6 ± 9.7 ^#^	73.3 ± 31.1	34.9 ± 22.1 ^#^
Total protein (g/L)	1000 IU	42.4 ± 1.5	43.7 ± 1.7	39.4 ± 3.3 ^#,&^	43.7 ± 1.0	40.8 ± 4.8
	1000 IU+cal	42.0 ± 3.3	46.1 ± 1.0^#^	41.2 ± 1.7^&^	43.0 ± 0.3	39.6 ± 4.0
	5000 IU	41.4 ± 2.4	45.5 ± 2.0 ^#^	41.5 ± 1.4 ^&^	45.2 ± 1.0	39.6 ± 5.8 ^#^
	100 IU	41.7 ± 1.0	43.6 ± 1.7	40.1 ± 1.7 ^&^	43.1 ± 0.1	40.0 ± 5.6
	100 IU+cal	44.4 ± 2.9	46.3 ± 2.9 *^,@^	42.2 ± 1.2 ^&^	43.7 ± 2.8	36.9 ± 7.0 ^#^
Albumin (g/L)	1000 IU	9.7 ± 1.6	10.8 ± 2.2	8.4 ± 2.7 ^&^	10.7 ± 2.1	10.3 ± 1.4
	1000 IU+cal	9.7 ± 3.0	11.1 ± 1.8	9.7 ± 1.7	13.0 ± 1.5 *	9.6 ± 2.8 ^#^
	5000 IU	10.1 ± 1.2	11.5 ± 4.0	10.6 ± 1.6	14.1 ± 2.0 *	10.1 ± 2.8 ^#^
	100 IU	9.9 ± 0.8	11.8 ± 1.0	9.9 ± 1.8	12.2 ± 2.8	9.9 ± 2.5
	100 IU+cal	11.7 ± 2.0	8.9 ± 1.8 ^#,@^	9.0 ± 1.4 ^#^	13.0 ± 1.2	9.2 ± 3.3 ^#^

N = 6 with exception for alkaline phosphatase: N = 5. Data are presented as mean with standard deviation. Statistical analysis: Sidak’s or Dunn’s multiple comparisons test. * *p* < 0.05 as compared to 1000 IU; ^#^ *p* < 0.05 tumor-bearing as compared to healthy mice from the same treatment group; ^&^ *p* < 0.05 67NR as compared to 4T1; ^@^ *p* < 0.05 as compared to 100 IU.

**Table 5:** Characteristics of white blood cells (WBCs) in BALB/c and C57BL/6 healthy and mammary gland tumor-bearing mice.

Blood Parameter	Group	Strain				
		BALB/c			C57BL/6	
		Healthy	4T1	67NR	Healthy	E0771
WBC (10^3^/µL)	1000 IU	4.7 ± 1.2	135.0 ± 71.2	6.6 ± 1.4	5.5 ± 0.9	6.8 ± 3.3
	1000 IU+cal	4.4 ± 1.3	181.0 ± 97.1	6.6 ± 0.9	6.0 ± 1.9 *	10.8 ± 6.5
	5000 IU	4.0 ± 0.9	199.4 ± 105.9	7.8 ± 1.9	5.6 ± 1.4	8.4 ± 5.1
	100 IU	3.8 ± 1.1 *	142.0 ± 92.0	7.1 ± 2.4	6.0 ± 1.2	12.1 ± 7.6 *
	100 IU+cal	3.9 ± 1.1	231.6 ± 136.8 *^,@^	5.8 ± 1.4	5.2 ± 0.9	11.1 ± 6.8 *
Lymph (10^3^/µL)	1000 IU	4.8 ± 1.2	31.4 ± 17.3	4.8 ± 1.1	5.1 ± 1.6	5.6 ± 3.2
	1000 IU+cal	4.4 ± 1.3	42.7 ± 23.9	5.2 ± 0.9	5.3 ± 1.7	9.2 ± 6.2
	5000 IU	4.0 ± 0.9	49.5 ± 28.1	6.1 ± 1.7	4.9 ± 1.2	7.2 ± 4.8
	100 IU	3.8 ± 1.1	38.1 ± 26.4 *	5.4 ± 1.8	4.9 ± 0.5	10.4 ± 7.2 *
	100 IU+cal	3.9 ± 1.1	62.3 ± 33.6 *^,@^	4.4 ± 1.1	4.4 ± 0.8	9.6 ± 6.0 *
Gran (10^3^/µL)	1000 IU	0.6 ± 1.7	79.7 ± 37.0	1.4 ± 0.5	0.3 ± 0.1	0.6±0.4
	1000 IU+cal	0.7 ± 0.3	99.9 ± 48.7	1.3 ± 0.2	0.3 ± 0.1	0.7 ± 0.5
	5000 IU	0.5 ± 0.2	107.6 ± 47.4	1.4 ± 0.4	0.3 ± 0.1	0.4 ± 0.2
	100 IU	0.5 ± 0.1	79.2 ± 42.1	1.4 ± 0.7	0.4 ± 0.2	0.6 ± 0.3
	100 IU+cal	0.5 ± 0.2	116.6 ± 54.2	1.2 ± 0.4	0.3 ± 0.1	0.4 ± 0.2
Mon (10^3^/µL)	1000 IU	0.3 ± 0.2	23.8 ± 18.3	0.3 ± 0.1	0.5 ± 0.2	0.8 ± 0.4
	1000 IU+cal	0.3 ± 0.2	38.3 ± 30.8	0.4 ± 0.2	0.3 ± 0.1	0.9 ± 0.6
	5000 IU	0.3 ± 0.1	35.2 ± 25.7	0.3 ± 0.1	0.4 ± 0.2	0.6 ± 0.3
	100 IU	0.2 ± 0.1	18.3 ± 13.2	0.2 ± 0.1	0.4 ± 0.2	0.9 ± 0.5
	100 IU+cal	0.2 ± 0.0	52.7 ± 52.5 *^,@^	0.2 ± 0.1	0.4 ± 0.2	0.9 ± 0.4

Blood collected from 10–12 mice/group was analyzed. Statistical analysis: Sidak’s test for multiple comparisons. Data underlined: *p* < 0.05 as compared to healthy mice from the same treatment group. * *p* < 0.05 as compared to 1000 IU; ^@^ *p* < 0.05 as compared to 100 IU.

## Supplementary Materials

The following are available online at https://www.mdpi.com/2072-6643/12/11/3416/s1, Figure S1: Nuclear expression of VDR in tumor and lung tissue. Immunohistochemical reactions. 4T1: (A) tumor tissue, (B) lung. 67NR: (C) tumor tissue, (D) lung. E0771: (E) tumor tissue, (F) lung. (G) Lung tissue from healthy C57BL/6 mouse. N = 5 mice/group. Magnification—×200, scale bars—50 µm. Statistical analysis: Dunn’s multiple comparisons test. * *p* < 0.05, Figure S2: Erythrocyte morphological analysis. MCV: mean cell volume; MCH: mean corpuscular hemoglobin; MCHC: mean corpuscular hemoglobin concentration; RDW: red distribution width. Statistical analysis: Dunn’s multiple comparisons test. * *p* < 0.05, Figure S3: Platelet numbers and other platelet parameter distributions in healthy and mammary gland tumor-bearing BALB/c mice. PCT: plateletcrit; PDV: platelet distribution width; MPV: mean platelet volume. Statistical analysis: Dunn’s multiple comparisons test. * *p* < 0.05, Figure S4: Selected blood morphological parameters of mice bearing E0771 mammary gland tumors. (A) Erythrocyte morphological parameters. (B) Platelet morphological parameters. MCV: mean cell volume; MCH: mean corpuscular hemoglobin; MCHC: mean corpuscular hemoglobin concentration; RDW: red distribution width. PCT: plateletcrit; PDV: platelet distribution width; MPV: mean platelet volume. Statistical analysis: Dunn’s multiple comparisons test. * *p* < 0.05, Figure S5: Proliferation inhibition of E0771 cells by calcitriol in vitro. A total of 10^4^ viable cells per well were plated in a 96-well plate. On the next day, calcitriol (1000, 100, 10, and 1 nM) and its solvent ethanol (1, 0.1, 0.01, and 0.001%) were applied for a 72-h incubation in triplicates. After this time, the sulforhodamine B (SRB) test was performed to determine the inhibition of proliferation. Absorbance was measured using a Synergy H4 plate reader (BioTek, Winooski, VT, USA) at a wavelength of 540 nm. The proliferation inhibition (%) was determined in relation to the untreated control cells. The test was repeated three times.
