# Supplementary material for: Vitamin D Metabolite Profile in Cholecalciferol- or Calcitriol-Supplemented Healthy and Mammary Gland Tumor-Bearing Mice
Source: Nutrients. 2020 Nov 6;12(11):3416. doi: 10.3390/nu12113416 (PMC7695033; doi:10.3390/nu12113416)
Supplement: Supplementary file 1 [file nutrients-12-03416-s001.pdf]

Supplementary material

Vitamin D Metabolite Profile in Cholecalciferol- or Calcitriol-Supplemented Healthy and Mammary Gland Tumor-Bearing Mice

Artur Anisiewicz [1], Konrad Kowalski [2], Joanna Banach [1], Natalia Łabędź [1], Martyna Stachowicz-Suhs [1], Aleksandra Piotrowska [3], Magdalena Milczarek [1], Dagmara Kłopotowska [1], Piotr Dzięgiel [3,4] and Joanna Wietrzyk [1,*]

[1] Department of Experimental Oncology, Hirszfeld Institute of Immunology and Experimental Therapy, 53-114 Wroclaw, Poland; artur.anisiewicz@hirszfeld.pl (A.A.); joanna.banach@hirszfeld.pl (J.B.); natalia.labedz@hirszfeld.pl (N.L.); martyna.stachowicz@hirszfeld.pl (M.S.-S.); magdalena.milczarek@hirszfeld.pl (M.M.); dagmara.klopotowska@hirszfeld.pl (D.K.)

[2] Research and Development Center Masdiag, 01-882 Warsaw, Poland; konrad.kowalski@mas-diag.pl

[3] Department of Histology and Embryology, Faculty of Medicine, Wroclaw Medical University, 50-368 Wroclaw, Poland; aleksandra.piotrowska@umed.wroc.pl (A.P.); piotr.dziegiel@umed.wroc.pl (P.D.)

[4] Department of Physiotherapy, Wroclaw University School of Physical Education, 51-612 Wroclaw, Poland

* Correspondence: joanna.wietrzyk@hirszfeld.pl; Tel.: +48-713-709-985

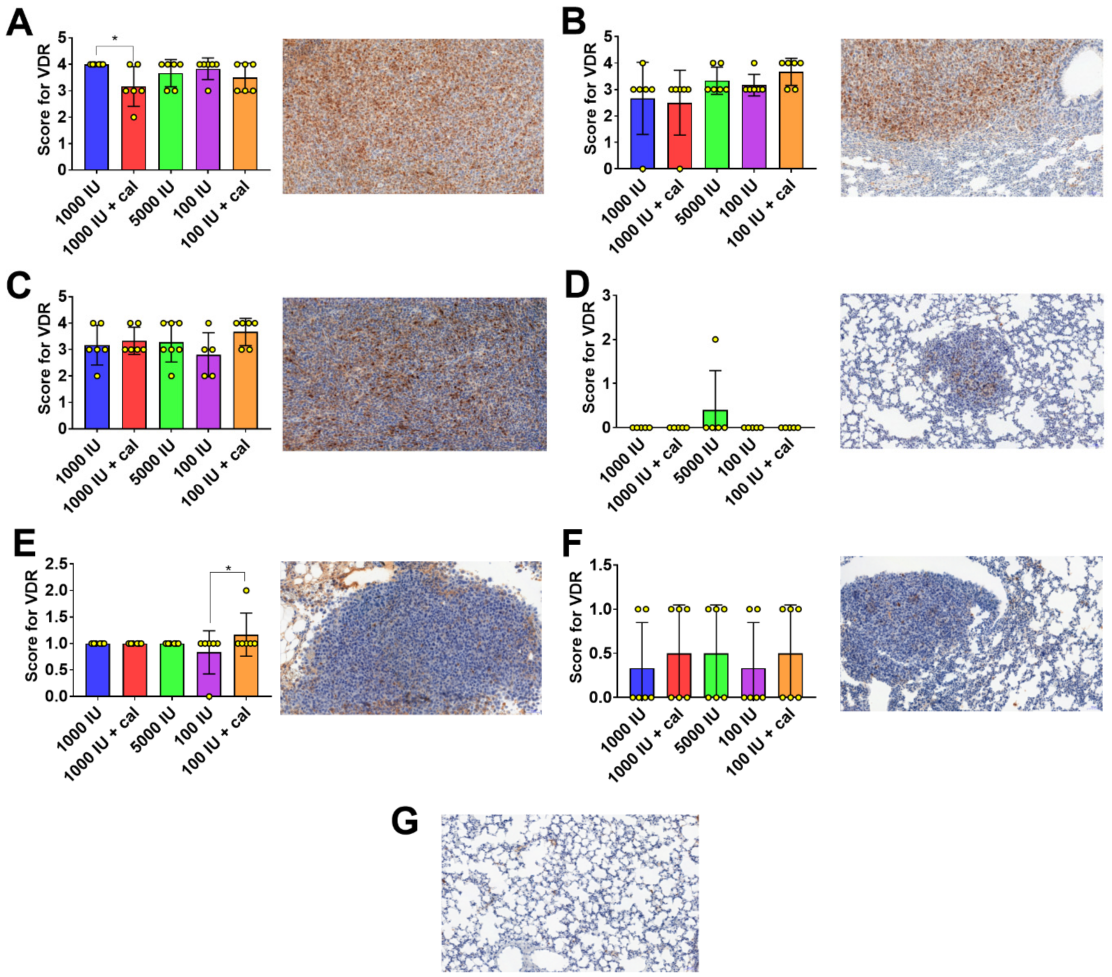

Figure S1: Nuclear expression of VDR in tumor and lung tissue. Immunohistochemical reactions. 4T1: (A) tumor tissue, (B) lung. 67NR: (C) tumor tissue, (D) lung. E0771: (E) tumor tissue, (F) lung. (G) Lung tissue from healthy C57BL/6 mouse. N = 5 mice/group. Magnification—×200, scale bars—50 μm. Statistical analysis: Dunn's multiple comparisons test. *$p < 0.05$

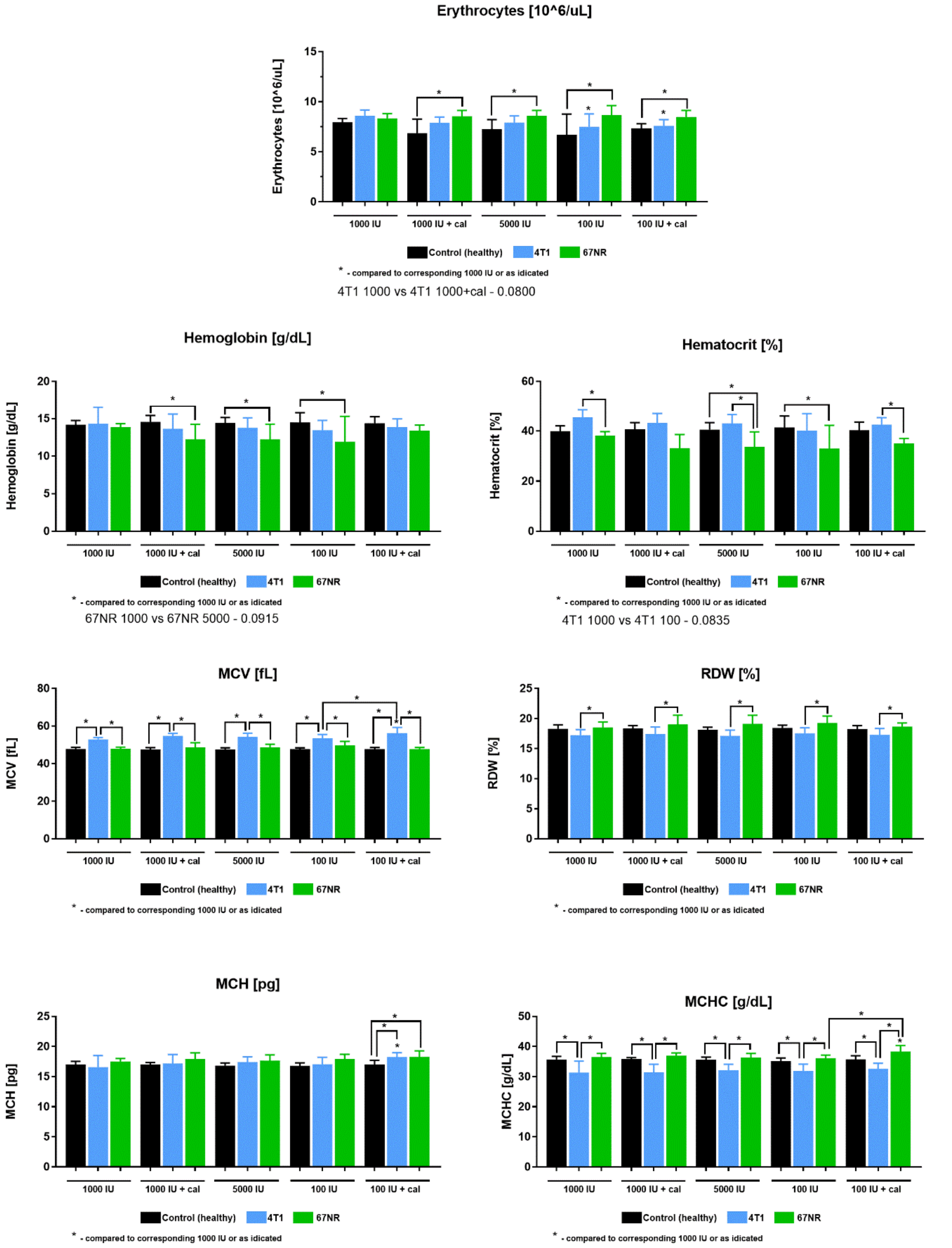

Figure S2: Erythrocyte morphological analysis. MCV: mean cell volume; MCH: mean corpuscular hemoglobin; MCHC: mean corpuscular hemoglobin concentration; RDW: red distribution width. Statistical analysis: Dunn's multiple comparisons test. *$p < 0.05$,

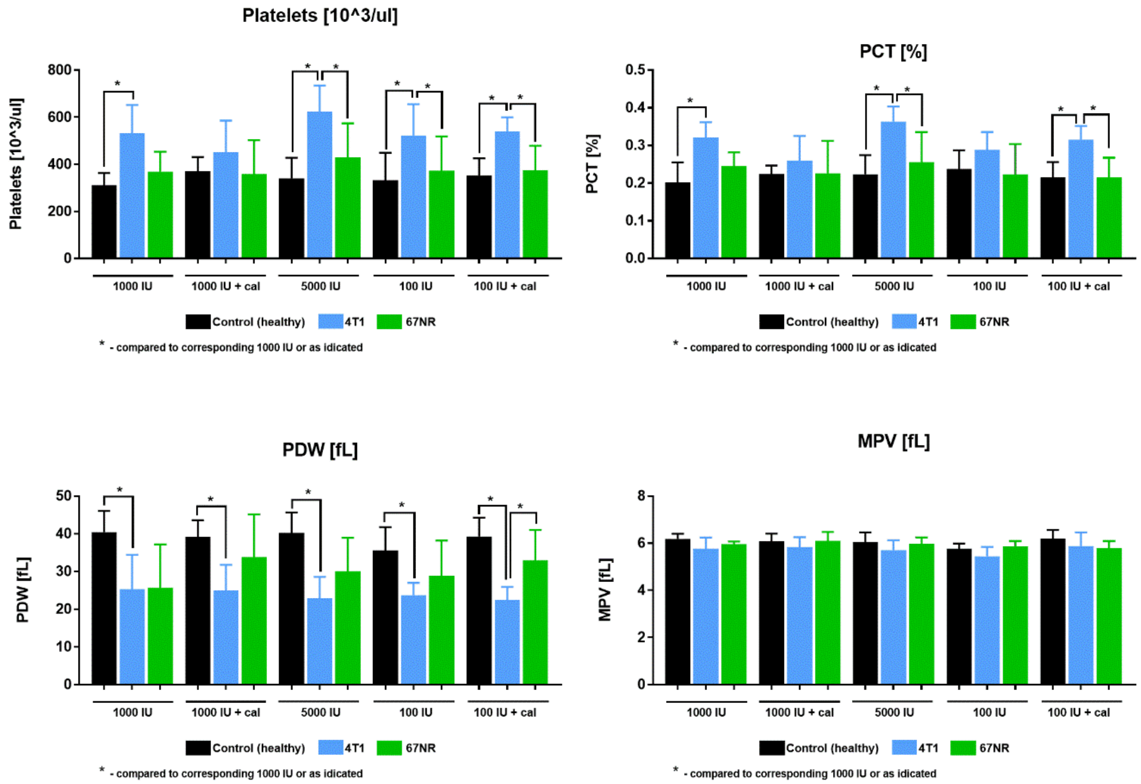

Figure S3: Platelet numbers and other platelet parameter distributions in healthy and mammary gland tumor-bearing BALB/c mice. PCT: plateletcrit; PDV: platelet distribution width; MPV: mean platelet volume. Statistical analysis: Dunn's multiple comparisons test. *$p < 0.05$

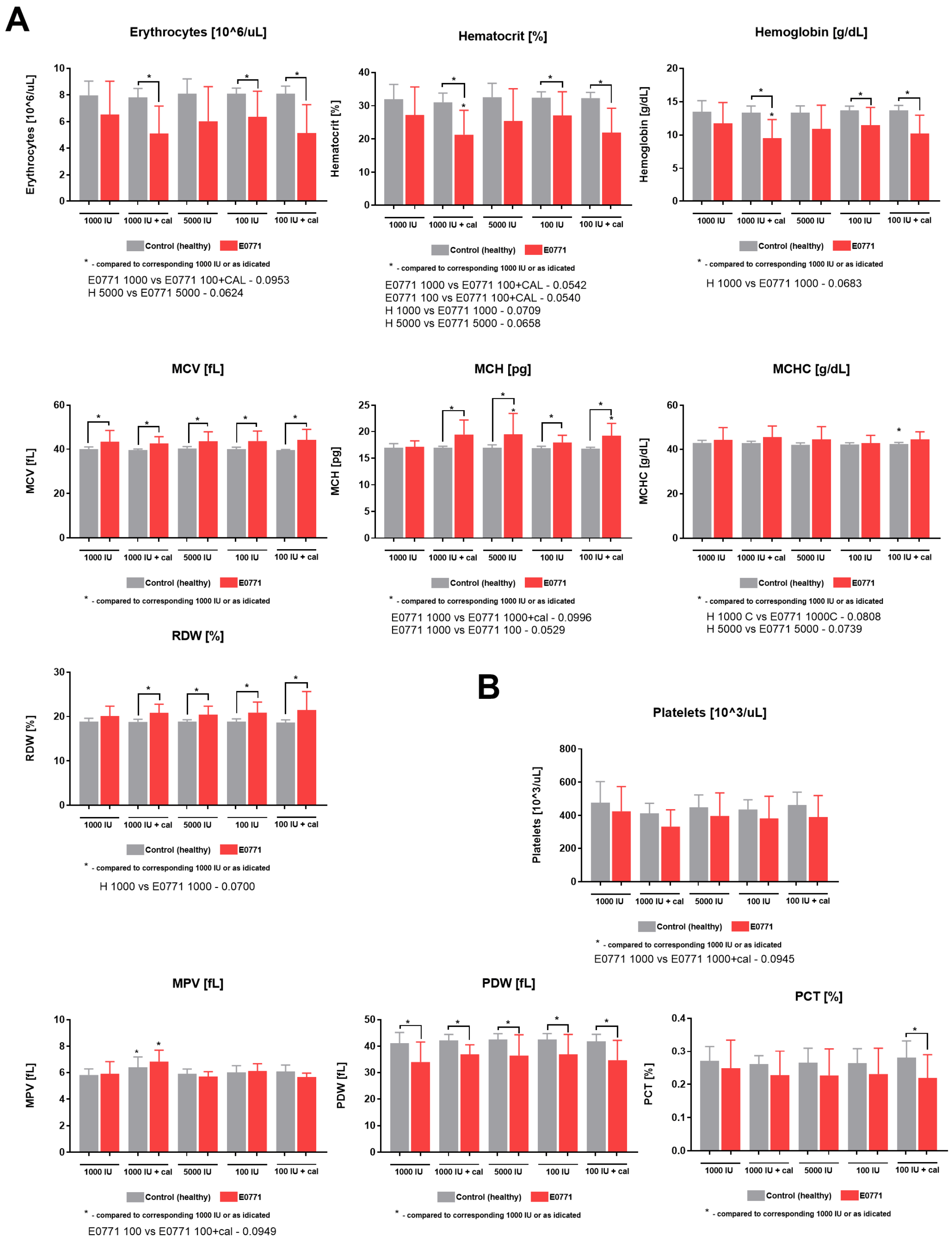

Figure S4: Selected blood morphological parameters of mice bearing E0771 mammary gland tumors. (A) Erythrocyte morphological parameters. (B) Platelet morphological parameters. MCV: mean cell volume; MCH: mean corpuscular hemoglobin; MCHC: mean corpuscular hemoglobin concentration; RDW: red distribution width. PCT: plateletcrit; PDV: platelet distribution width; MPV: mean platelet volume. *Statistical analysis: Dunn's multiple comparisons test. *$p < 0.05$

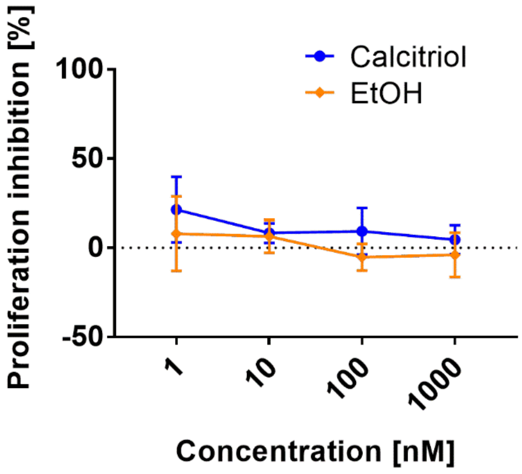

Figure S5: Proliferation inhibition of E0771 cells by calcitriol in vitro. A total of 10^4 viable cells per well were plated in a 96-well plate. On the next day, calcitriol (1000, 100, 10, and 1 nM) and its solvent ethanol (1, 0.1, 0.01, and 0.001%) were applied for a 72-h incubation in triplicates. After this time, the sulforhodamine B (SRB) test was performed to determine the inhibition of proliferation. Absorbance was measured using a Synergy H4 plate reader (BioTek, Winooski, VT, USA) at a wavelength of 540 nm. The proliferation inhibition (%) was determined in relation to the untreated control cells. The test was repeated three times
